# Proximal tubule ACE2 mediates early responses to hypertension by regulating intrarenal RAS and sodium homeostasis

**DOI:** 10.1172/jci.insight.179960

**Published:** 2026-09-22

**Authors:** Jacqueline M. Emathinger, Zhidan Xiang, Jonathan W. Nelson, Matthew W. Hagen, Nakyung Kim, David I. Ortiz-Melo, Natalie Mattocks, Jorge F. Giani, Dexter L. Lee, Hannah Hartman-Houstman, Donna L. Ralph, Alicia A. McDonough, Brianna Chen, Stan G. Louie, Thomas M. Coffman, Susan B. Gurley

**Affiliations:** 1 Division of Nephrology and Hypertension, Department of Medicine, Oregon Health & Science University, Portland, Oregon, USA.; 2 Division of Nephrology, Department of Medicine, Duke University and Durham VA Health Care Centers, Durham, North Carolina, USA.; 3 Department of Neurosurgery, Wake Forest School of Medicine, Winston-Salem, North Carolina, USA.; 4 Division of Nephrology and Hypertension, Department of Medicine, Keck School of Medicine, University of Southern California, USA.; 5 Department of Biomedical Sciences, Cedars-Sinai Medical Center, Los Angeles, California, USA.; 6 Department of Physiology and Biophysics, College of Medicine, Howard University, Washington, DC, USA.; 7 Department of Physiology and Neuroscience, Keck School of Medicine, and; 8 Clinical Pharmacy and Experimental Therapeutics Program, Alfred E. Mann School of Pharmacy and Pharmaceutical Sciences, University of Southern California, Los Angeles, California, USA.; 9 Program in Cardiovascular & Metabolic Disorders, Duke-NUS Medical School, Singapore.

**Keywords:** Cardiology, Nephrology, Hypertension, Mouse models, Sodium channels

## Abstract

ACE2 is a membrane-bound monocarboxypeptidase strongly expressed in the renal proximal tubule (PT) with high affinity to degrade the vasopressor angiotensin II (AngII). We employed a mouse model of PT-specific ACE2 deletion (PT ACE2–KO) to demonstrate that the renal PT is a critical site for ACE2 regulation of blood pressure (BP) via modulation of the intrarenal renin-angiotensin system (RAS). While deletion of ACE2 from the PT had a minimal effect on baseline physiology, PT ACE2–KO mice were more susceptible to AngII hypertension than control mice. At day 5 of AngII infusion, the enhanced BP response was associated with cardiac hypertrophy, increased renal AngII levels, failure to suppress epithelial sodium channel (ENaC) γ cleavage, and increased sodium pump activity in PT ACE2–KO mice. Control mice instead increased renal ACE2 expression to reduce renal AngII accumulation and suppress intrarenal RAS activation, which offered protection from hypertension and complications. Transcriptional analysis corroborated changes in intrarenal RAS components and revealed alterations in distinct physiological pathways during AngII hypertension in PT ACE2–KO mice. Our studies provide evidence for alterations in ENaC regulation to contribute to the development of AngII hypertension and support PT-derived ACE2 as an integral member of the intrarenal RAS.

## Abstract 2

Our study shows how ACE2 regulates the kidney’s local renin-angiotensin system and salt balance, revealing new insights into how hypertension develops.

## Introduction

Hypertension is the leading cause of cardiovascular disease and preventable death globally (1, 2). A primary determinant of systemic blood pressure (BP) is extracellular fluid volume, which is controlled by the kidney. The renal proximal tubule (PT) is the initial site in the nephron where two-thirds of glomerular filtrate is reabsorbed, and disruption of the primary functions of the PT is a known mechanism underlying hypertension (3). Increasing sodium reabsorption in the PT increases extracellular fluid volume, which elevates BP, a signal to then reduce proximal nephron sodium reabsorption to maintain fluid homeostasis. Many of these functions are regulated by the renin-angiotensin system (RAS) through the actions of the biologically active peptide, angiotensin II (AngII). In addition to systemic vasoconstriction, AngII acts locally within the kidney via its receptors to activate the intrarenal RAS and promote sodium retention via regulation of solute transporters in the PT and other nephron segments, together altering set point for pressure natriuresis and increasing BP (4–6). Reductions in AngII levels resulting from loss of renal angiotensin-converting enzyme (ACE), which catalyzes formation of AngII (7), and reduced AngII signaling via type 1 angiotensin receptors in the PT (5) both blunt hypertension. An additional path to oppose the actions of AngII involves the monocarboxypeptidase ACE2, which degrades AngII to form angiotensin 1–7 (Ang[1–7]) (8, 9). Within the kidney, ACE2 expression is most robust on the apical surface of the PT, where it is poised to oppose the actions of ACE and reduce AngII levels to restrict its actions locally. Due to its ability to counterbalance the actions of AngII, ACE2 has been studied as a potential antihypertensive agent and treatment for diabetic kidney disease (10–15). More recently, ACE2 has been the focus of therapeutic approaches to prevent severe acute respiratory syndrome coronavirus-2 (SARS-CoV-2) infection and complications as ACE2 serves as the receptor for SARS-CoV-2 (16).

Since its first description in 2000 (9), the overall significance of ACE2 within the local renal and systemic RAS has been supported by numerous studies. Our group generated one of the original ACE2-KO mouse lines and demonstrated that global deletion of ACE2 increased susceptibility to AngII-mediated hypertension and was associated with accumulation of AngII peptides locally within the kidney (5). Loss of ACE2 exacerbated renal and cardiac dysfunction, as manifested through increased renal oxidative stress (17–19), worsening of diabetic nephropathy (20, 21), and development of cardiac hypertrophy and contractile dysfunction (17, 22). Conversely, overexpression of ACE2 in the central nervous system was protective against hypertension and cardiac hypertrophy (23–25), while use of recombinant ACE2 has been shown to confer antihypertensive and renal-protective effects in vivo (11, 26). Taken together, these studies demonstrate that ACE2 plays a critical role in the maintenance of renal and cardiovascular physiology, yet the effect of these actions and precise cellular locations where they occur have not been described.

We previously performed kidney cross-transplantation studies between ACE2-KO and WT littermate mice to isolate renal and extrarenal (systemic) sources of ACE2 (27). These studies unexpectedly revealed that cleavage of the ACE2 ectodomain from extrarenal sources yielded a catalytically active soluble ACE2 (sACE2) fragment, which could be filtered by the glomerulus to reach the lumen of the nephron to protect against AngII-mediated hypertension (9, 17, 19). Thus, filtration of systemically derived sACE2 represents an additional means by which the intrarenal RAS can be regulated. The potential for ACE2 to modulate the RAS in local and distant sites is further suggested by work from Lazartigues’ group studying neurogenic hypertension, in which shedding of sACE2 displaces the enzyme from its site of action in the central nervous system resulting in impaired AngII metabolism in the brain (28). Thus, ACE2 cleavage can serve to relocate its enzymatic action and alter RAS activity depending upon whether sACE2 either gains access to or is removed from AngII tissue targets involved in disease pathology. ACE2 cleavage has also been implicated in the pathogenesis of SARS-CoV-2 as viral binding to ACE2 induces shedding of the ectodomain portion while allowing for viral entry into cells. In patients with recent SARS-CoV-2 infection, viral binding-mediated loss of tissue ACE2 may drive new-onset hypertension (16, 29–33). These data emphasize the importance and complexity of ACE2 in disease states.

Our present studies examine the physiological roles of PT ACE2 through the generation of a mouse line lacking ACE2 specifically in the PT (PT ACE2 KO). This mouse model allows us to pinpoint the location and functions of a key RAS regulator by targeting the renal PT, the nephron segment recognized for primacy of sodium transport (3), marked abundance of ACE2, and prominence of AngII signaling for BP control (4, 5, 34). In addition to detailed cardiovascular assessments, we combine quantitative immunoblotting of key sodium transporters with evaluation of transcriptional changes of solute transporters and pathway enrichment analysis using single-nucleus RNA-seq (snRNA-seq) to define how loss of ACE2 from the PT affects function along the entire nephron. Our work demonstrates that PT-derived ACE2 acts as a critical buffer against intrarenal AngII accumulation, helps to preserve the pressure natriuretic response, and reduces hypertensive complications.

## Results

### PT-specific deletion of ACE2.

**Figure 1:**
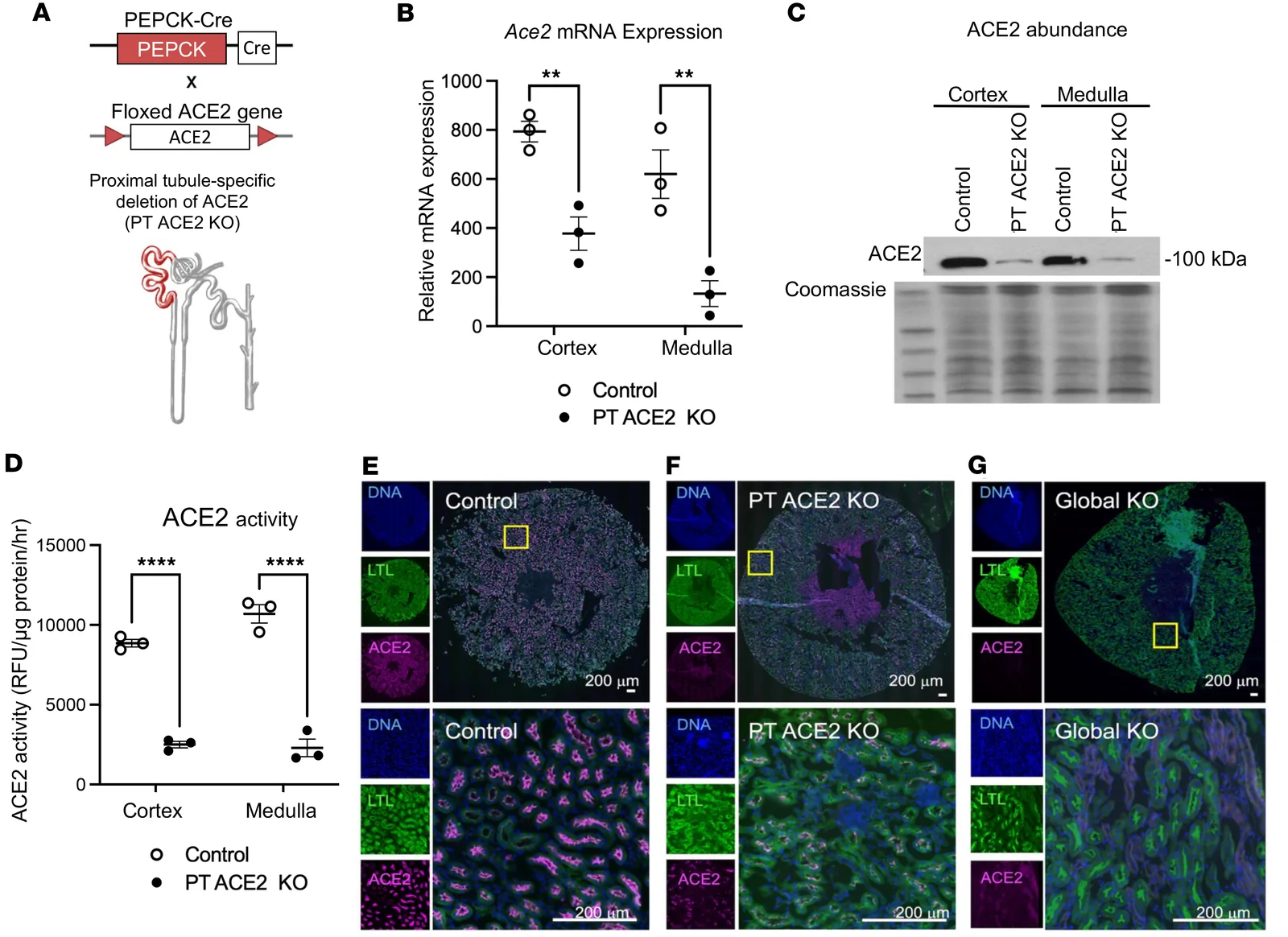
Proximal tubule-specific deletion of ACE2. (**A**) PT ACE2–KO mice were generated by crossing mice expressing Cre-recombinase downstream of the PEPCK promoter with ACE2-floxed mice. (**B**–**D**) *Ace2* mRNA expression, ACE2 protein abundance and Coomassie brilliant blue loading control, and ACE2 activity in kidney cortex and medulla of PT ACE2–KO and Control mice. (**E**) IHC for ACE2 in the proximal tubule in Control kidneys. (**F**) ACE2 expression is decreased in PT ACE2–KO mice (representative images; *n* = 3 mice per group). (**G**) ACE2 is absent in Global KO mice. Hoechst DNA stain in blue, Lotus Tetragonolobus Lectin (LTL) proximal tubule marker in green, and ACE2 in magenta. Scale bar: 200 μm. Numeric data expressed as mean ± SEM and analyzed as a 2-way ANOVA with Šídák’s multiple-comparison post hoc analysis. *n* = 3 mice per group, **P < 0.01, ****P < 0.0001.

Mice lacking ACE2 specifically in the renal PT (PT ACE2–KO mice) were generated by crossing mice expressing Cre-recombinase under a modified PEPCK promoter (5, 35) with mice bearing a conditional “floxed” *Ace2* allele previously generated by our laboratory (Figure 1A) (36). We verified specificity of gene deletion in the cell-specific deletion model compared with littermate Control mice bearing only the conditional allele, and we found significant loss of ACE2 expression in both the renal cortex and medulla of PT ACE2–KO mice by qPCR (Figure 1B), immunoblotting (Figure 1C), and ACE2 enzymatic activity assay (Figure 1D). To confirm cell specificity of reductions in ACE2 expression, we employed immunofluorescence and compared kidney sections from PT ACE2–KO, Control, and global ACE2-KO mice (Figure 1, E–G), including costaining with lotus tetragonolobus lectin (LTL), a PT-specific marker. Despite robust genetic deletion, PT ACE2–KO mice appeared healthy with expected gene transmission rates. As both targeted genes are on the X chromosome, only male mice aged 8–26 weeks were used in this study.

### PT-derived ACE2 attenuates early hypertension and cardiac hypertrophy.

**Figure 2:**
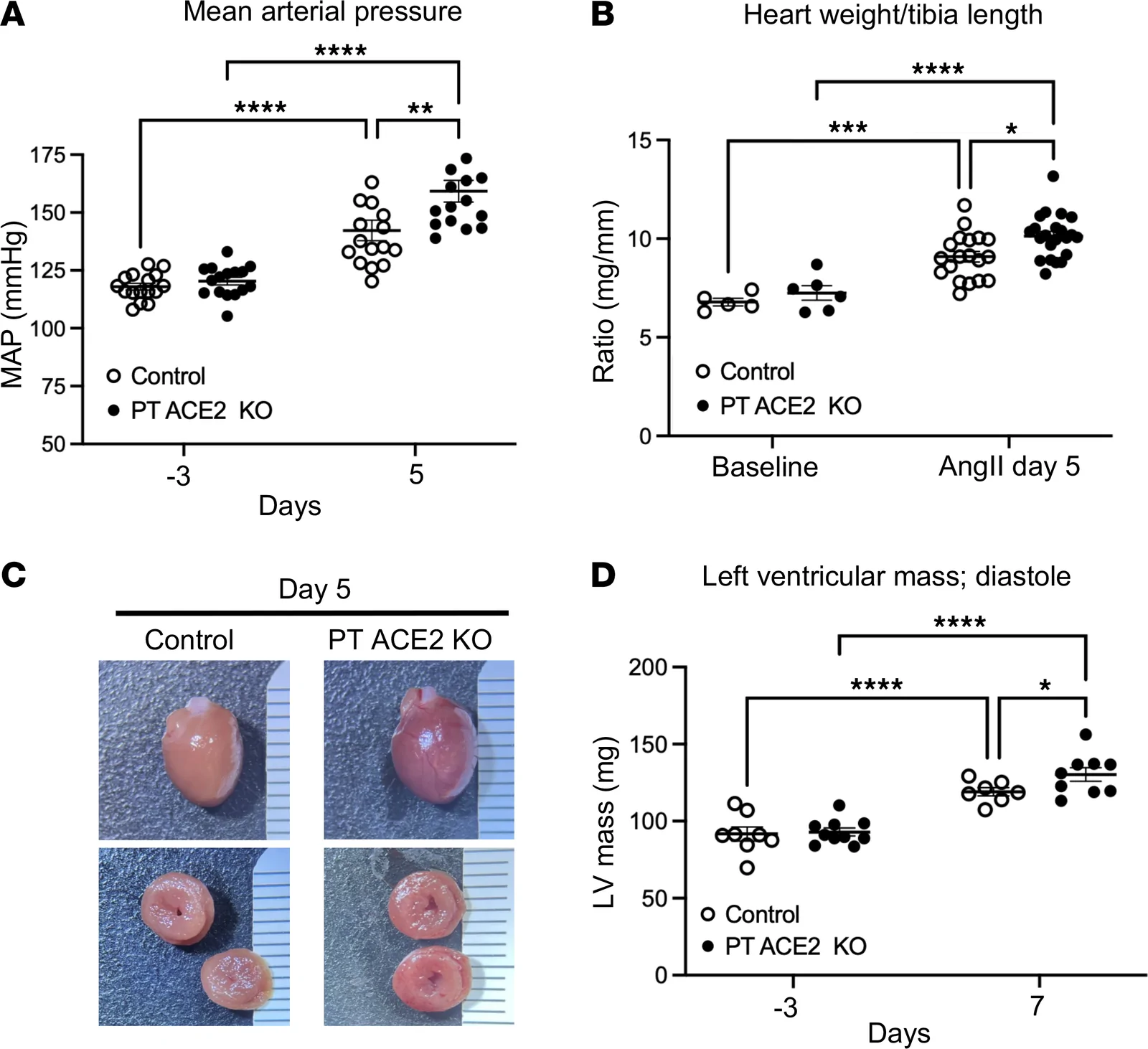
Proximal tubule ACE2 attenuates early hypertension and cardiac hypertrophy. (**A**) Mean arterial pressures (MAP) at baseline (day –3) and with continuous AngII infusion (1,000 ng/kg/min) at day 5. Day 5 MAP values are also presented in Figure 3A. (**B**) Ratio of heart weight to tibia length (HW/TL) in mice at baseline and treated with AngII for 5 days (*n* = 5–6 for baseline groups; *n* = 19–23 for AngII treated groups; figure displays aggregated data across multiple experiments). (**C**) Representative anterior-view and mid-ventricular cross-sections of hearts from Control and PT ACE2–KO mice after 5 days of AngII treatment (*n* = 8–10 mice per group). (**D**) Echocardiographic calculated left ventricular (LV) mass at diastole (*n* = 8–10 mice per group). Data expressed as mean ± SEM. MAP and HW/TL data were analyzed using a 2-way ANOVA with Tukey’s multiple-comparisons post hoc analysis. Echocardiography data was analyzed by mixed-effects analysis with Fisher’s LSD pairwise comparison post hoc analysis. *P < 0.05, **P < 0.01, ***P < 0.001, ****P < 0.0001.

**Figure 3:**
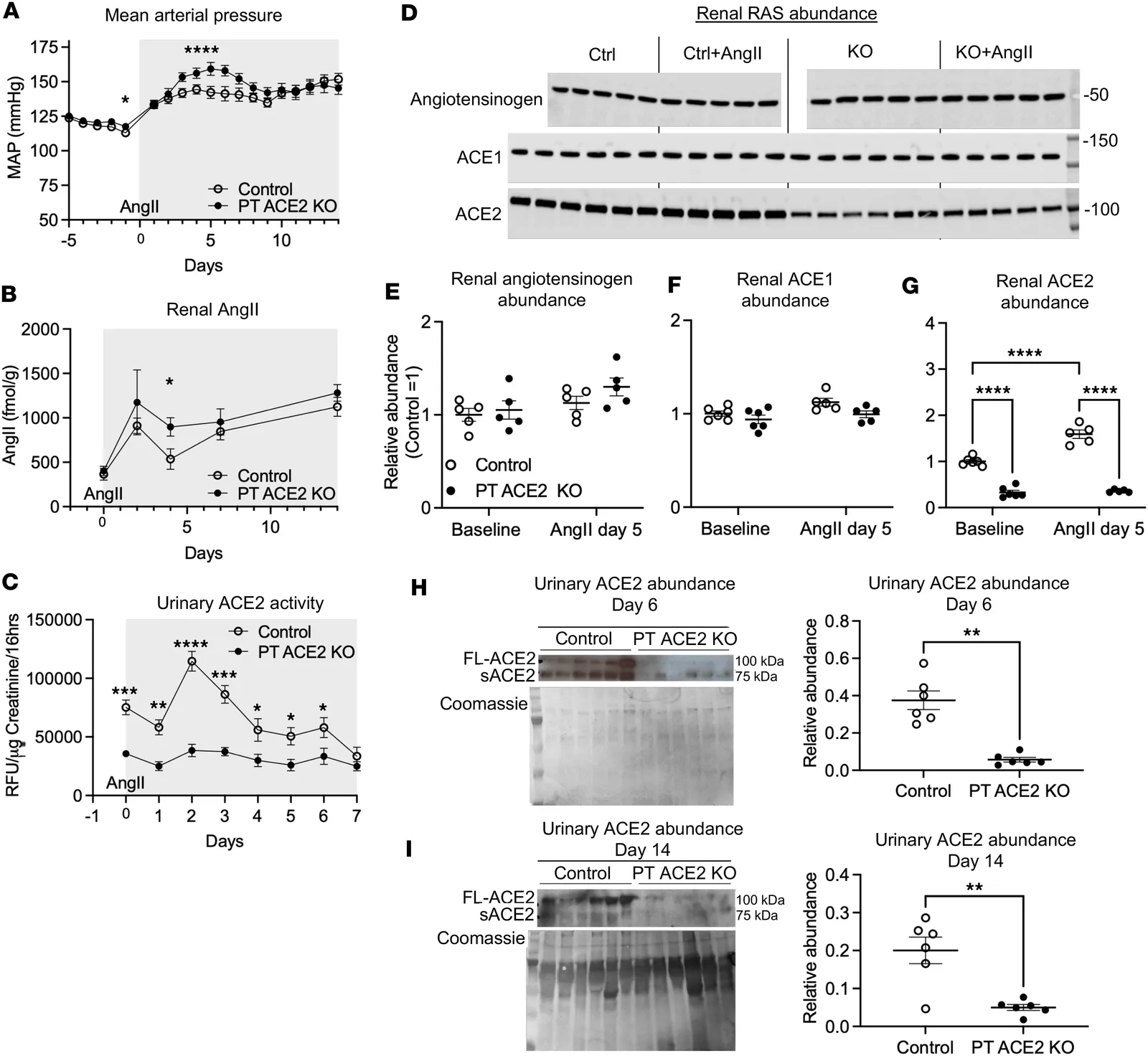
Proximal tubule ACE2 stabilizes response to AngII by mitigating increases in intrarenal RAS activity. (**A**) Mean arterial pressure (MAP) curve at baseline and with continuous AngII infusion (1,000 ng/kg/min) for 15 days. Day 5 MAP values are also presented in Figure 2A. (**B**) Renal AngII levels from Control and PT ACE2–KO mice. (**C**) Urinary ACE2 activity. (**D**) Immunoblots of angiotensinogen (*n* = 5), ACE1 (*n* = 6), and ACE2 (*n* = 6) protein abundance at baseline and with 5 days of AngII infusion. All lanes of the angiotensinogen immunoblot were run on the same gel but were noncontiguous. (**E**–**G**) Quantification of renal angiotensinogen, ACE1, and ACE2 abundance. (**H** and **I**) Immunoblots depicting urinary abundance of full-length ACE2 (FL-ACE2) and soluble ACE2 (sACE2) at day 6 and 14 days of AngII treatment. Coomassie brilliant blue stain was used to demonstrate similar loading for urine blots (56, 62). Data expressed as mean ± SEM. MAP (*n* = 16) was analyzed by mixed-effects analysis with Fisher’s LSD pairwise comparison post hoc analysis. Renal AngII (*n* = 3–10), urinary ACE2 activity (*n* = 6), and renal protein abundance were analyzed using a 2-way ANOVA with Tukey’s post hoc multiple comparison. Urinary ACE2 levels were analyzed by an unpaired 2-tailed *t* test with Welch’s correction (**H** and **I**). *P < 0.05, **P < 0.01, ***P < 0.001, ****P < 0.0001.

We evaluated the effect of PT ACE2 deletion on BP regulation using continuous radiotelemetry. Baseline BPs were similar between Control and PT ACE2–KO mice except at day –1, where PT ACE2–KO mice had higher BP (117.5 ± 6.4 mmHg versus 113.0 ± 5.2 mmHg, *P* < 0.05). BPs increased significantly during AngII infusion in both groups. However, by day 3 of continuous AngII infusion PT ACE2–KO mice had significantly higher mean arterial pressures (MAPs) relative to Control, peaking at day 5 (159.3 ± 18.7 mmHg in PT ACE2–KO versus 142.3 ± 17.6 mmHg in Control, *P* = 0.01; Figure 2A, Figure 3A, and Table 1). To evaluate the effect of enhanced hypertension in PT ACE2–KO mice, we assessed the cardiac hypertrophic response to AngII, using heart weight normalized to tibia length (HW/TL). Measurement of HW/TL is optimal for evaluation of cardiac hypertrophy in mice, as it is not affected by variability in animal weight during the experimental period (37). Corresponding to the increased MAPs at day 5, HW/TL was elevated in both groups but was significantly higher in PT ACE2–KO mice (10.13 ± 1.1 mg/mm versus 9.09 ± 1.2 mg/mm, *P* = 0.004; Figure 2, B and C). Echocardiography measurements of left ventricular (LV) mass corroborated the increase in heart weight in both groups, with a significant difference in LV mass seen between PT ACE2-KO and Control mice after 7 days of AngII treatment (130.3 ± 13.2 mg in PT ACE2–KO and 119.1 ± 7.2 mg in Control, *P* = 0.048; Figure 2D). Loss of ACE2 from the PT alone is sufficient to increase susceptibility to hypertension and cardiac hypertrophy early during AngII infusion.

### PT ACE2 stabilizes response to AngII by mitigating increases in intrarenal RAS activity.

PT ACE2–KO mice developed higher elevation of MAPs during the initial days of AngII treatment compared with Control mice, with statistically significant differences between groups at days 3, 4, 5, and 6 (Figure 2A and Figure 3A). Increased renal AngII levels were seen in PT ACE2–KO mice throughout this period with the biggest difference on day 4, corresponding to the timing of exaggerated BP response to AngII (day 4 AngII: 896.7 ± 330.4 fmol/g in PT ACE2–KO versus 536.5 ± 325.2 fmol/g in Control, *P* = 0.04; Figure 3B). In PT ACE2–KO kidneys, renal cortical abundance of angiotensinogen tended to be higher during AngII infusion compared with baseline by approximately 30% (1.3 ± 0.22; Figure 3, D and E), but this did not reach statistical significance. No differences in ACE1 abundance were detected in either Control or PT ACE2–KO mice (Figure 3, D and F). Control mice increased renal ACE2 protein levels by nearly 60% from baseline (1.59 ± 0.04-fold, *P* < 0.05; Figure 3, D and G), whereas ACE2 levels remained unchanged in PT ACE2–KO mice, as expected.

To investigate the effect of PT ACE2 deletion on systemic RAS tone, we assessed plasma ACE2 enzymatic activity, kidney renin expression, urinary aldosterone excretion, and circulating RAS metabolites in PT ACE2–KO versus Control mice after 5 days of AngII infusion (Supplemental Figure 1, A–C, and Supplemental Table 2; supplemental material available online with this article; https://doi.org/10.1172/jci.insight.179960DS1), as previously described (38). Global *Ace2* deletion has previously been shown to alter circulating AngII (Ang[1-8]) levels (39). However, in PT ACE2–KO and Control mice we observed no significant differences in plasma ACE2 activity, kidney *Ren1* expression, aldosterone excretion, or in circulating RAS metabolites. Although Ang(1–7) levels were not altered, the formation of Ang(1–9) from Ang(1–10) appears lower in the PT ACE2–KO mice, trending toward significance (Supplemental Table 2). The ratio of Ang(1–9)/Ang(1–10) — product over reactant catalyzed by ACE2 — was 6-fold higher in the Control as compared with PT ACE2 KO (*P* = 0.08). While these findings do not conclusively demonstrate alterations in the systemic RAS, the observed trends across multiple RAS components support an overall reduction of systemic RAS activity. These findings suggest that PT ACE2 deletion predominantly affects the intrarenal RAS, and that this is sufficient to influence HTN in PT ACE2–KO mice (Figure 2A and Figure 3A) (5, 40). To determine whether fluctuations in urinary ACE2 abundance underlie the temporal pattern of BP differences, we assessed urinary ACE2 activity (Figure 3C) and protein levels (Figure 3, H and I) in PT ACE2–KO and Control mice after 6 days or 14 days of AngII infusion. As shown in Figure 3C, urinary ACE2 activity increased significantly by approximately 52% in Control mice during the initial 3 days of AngII infusion (day 2 versus day 0, *P* < 0.05), suggesting an acute compensatory response to AngII infusion. By contrast, urinary ACE2 activity did not increase during the first week of AngII infusion in PT ACE2 KOs even during this period of transiently higher MAPs and AngII peptide levels. After day 2 of AngII infusion, urinary ACE2 activity fell progressively in Control mice such that, by day 7, it resembled the consistent, low levels seen in PT ACE2 KOs, demonstrating that AngII infusion induces an acute upregulation of ACE2 in Control mice, potentially as a mechanism to protect against BP elevation.

Previous studies have shown that both 98 kDa (“full-length”) and 75 kDa (“soluble”) ACE2 fragments can be detected in urine, and sACE2 can be filtered by the glomerulus to reach the lumen of the nephron (27), where it influences the activity of the intrarenal RAS. Thus, urinary ACE2 protein and activity levels may reflect the combined activities of full-length and sACE2. To delineate the relative contributions of PT-derived and systemic-derived sACE2 to urinary ACE2 protein, we probed for ACE2 in urine by immunoblotting with an antibody targeted to the N-terminus, which could distinguish the 2 isoforms by size. After 6 days of AngII treatment, both the 98 kDa full-length and 75 kDa sACE2 fragments were observed in urine of Control animals (Figure 3H). By contrast, only modest amounts of sACE2 and low levels of full-length ACE2 were observed in urine from PT ACE2–KO mice. Later, after 14 days of AngII infusion, levels of both full-length ACE2 and sACE2 were low in Control urine, consistent with the observed reduction in urinary ACE2 activity over time (Figure 3I). By 14 days, sACE2 was minimally detected in the urine of PT ACE2–KO mice. Taken together, these findings identify the PT as the major source of both full-length and sACE2 in urine, and we suggest that these moieties contribute to the antihypertensive actions of ACE2 in the early phase of AngII hypertension. Altogether, the increases in renal and urinary ACE2 in Control mice during the first week of AngII infusion appear to mitigate intrarenal RAS activity, corresponding with lower BP.

### Electrolyte homeostasis in PT ACE2–KO mice.

As our studies target the renal PT, the major site of sodium reabsorption by the nephron, we performed a balance study to determine whether the early hypertensive response seen in PT ACE2–KO mice was associated with altered sodium handling. No difference in urinary sodium excretion (UNaV) or sodium balance was observed between PT ACE2–KO and Control mice during the first 5 days of AngII treatment (Supplemental Figure 2, A and D) or in response to an acute saline challenge (Supplemental Table 3). Potassium balance was also similar in the 2 groups (Supplemental Figure 2, B and E). While we did not detect differences in creatinine clearance (CCr), a marker for GFR, or blood urea nitrogen during AngII treatment in either PT ACE2 KO or Control mice (Supplemental Figure 2, F and G), there was a modest trend toward increased CCr in PT ACE2–KO mice under baseline conditions, suggesting a role for PT-derived ACE2 in modulating renal function. There was an increase in albuminuria in PT ACE2–KO mice at this time point (Supplemental Figure 2H), consistent with their elevated BP and LV hypertrophy. As outlined below, the similarities in sodium and potassium balance may be explained by our sodium transporter studies, which reveal independent molecular changes aimed at maintaining sodium homeostasis.

### Alterations in sodium transporters along the nephron.

**Figure 4:**
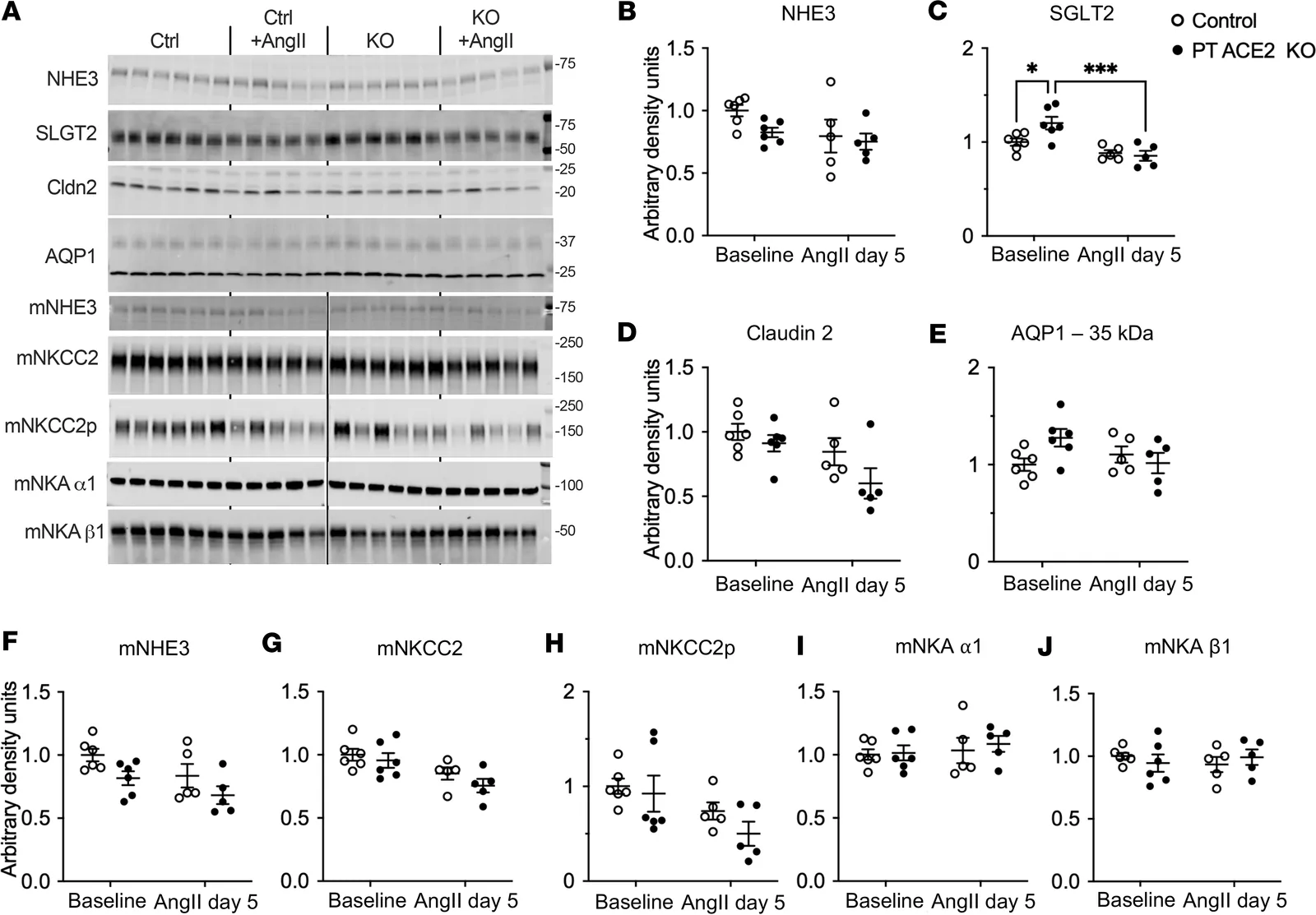
Alterations in sodium transporters along the proximal tubule and medullary thick ascending limb. (**A**) Immunoblots depicting key sodium transporter abundance at baseline and with 5 days of AngII. (**B**) Sodium hydrogen exchanger 3 (NHE3). (**C**) Sodium-glucose cotransporter 2 (SGLT2). (**D**) Claudin 2. (**E**) Aquaporin 1 major channel (AQP1-35 kDa). (**F**) Medullary NHE3. (**G**) Medullary sodium-potassium-chloride cotransporter (mNKCC2). (**H**) Phosphorylated mNKCC2. (**I**) Na^+^/K^+^ ATPase (NKA), α 1 subunit. (**J**) NKA β 1 subunit. All lanes of the immunoblots for mNHE3, mNKCC2, mNKCC2p, mNKA α1, and mNKA β1 were run on the same gel but were noncontiguous (*n* = 5–6 mice per group). Transporter abundance was analyzed as a 2-way ANOVA with Tukey’s multiple-comparison post hoc analysis. *P < 0.05, ***P < 0.001.

**Figure 5:**
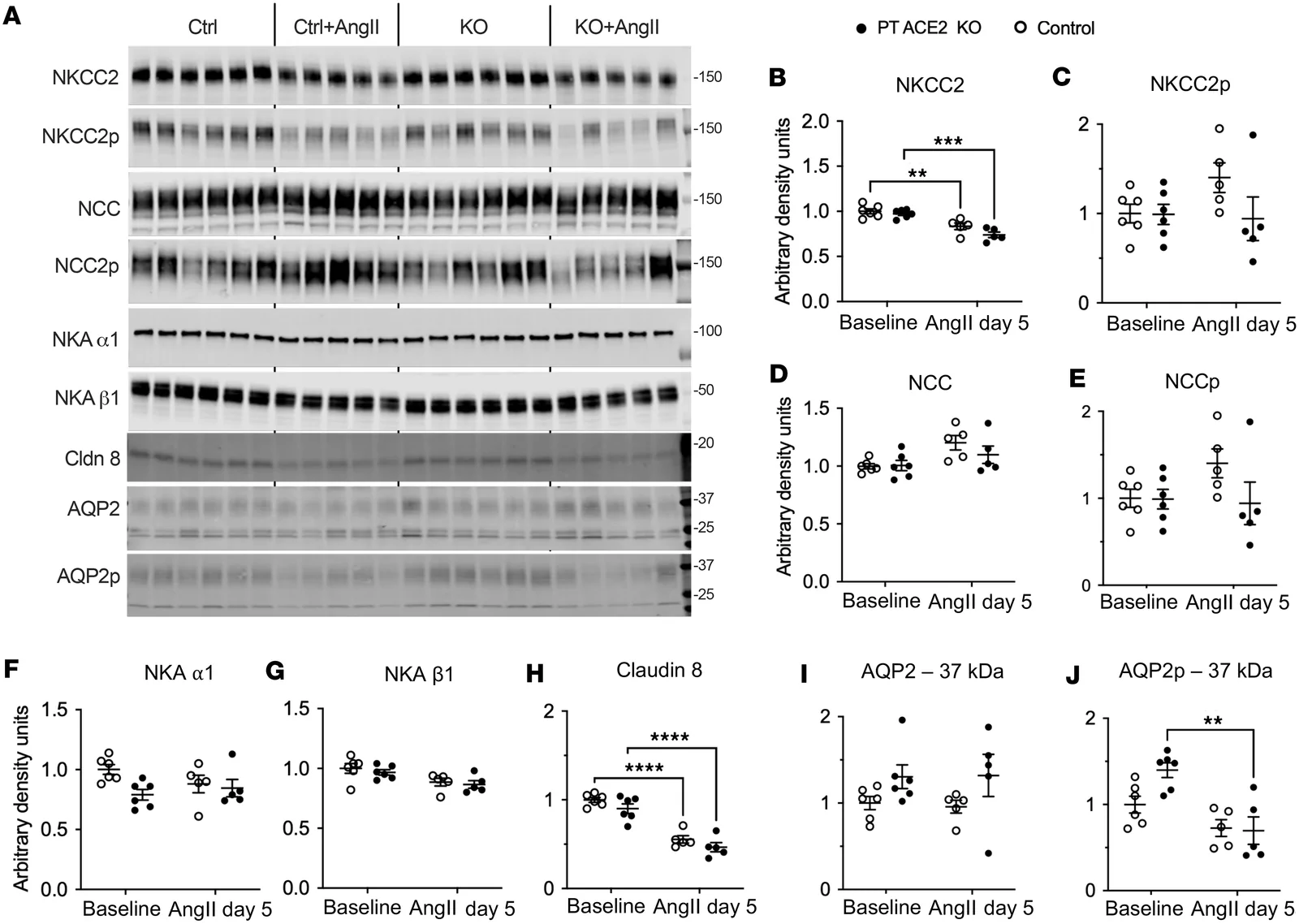
Alterations in sodium transporters along the more distal segments of the nephron. (**A**) Immunoblots depicting key sodium transporter abundance at baseline and with 5 days of AngII. (**B**) Sodium-potassium-chloride cotransporter (NKCC2). (**C**) Phosphorylated NKCC2. (**D**) Sodium-chloride cotransporter (NCC). (**E**) Phosphorylated NCC. (**F**) Na^+^/K^+^ ATPase (NKA), α1 subunit. (**G**) NKA β1 subunit. (**H**) Claudin 8 (Cldn 8). (**I**) Aquaporin 2 (AQP2), major band (37 kDa). (**J**) Phosphorylated AQP2 major band. Transporter abundance was analyzed as a 2-way ANOVA with Tukey’s multiple-comparison post hoc analysis (*n* = 5–6 mice per group). **P < 0.01, ***P < 0.001, ****P < 0.0001.

**Figure 6:**
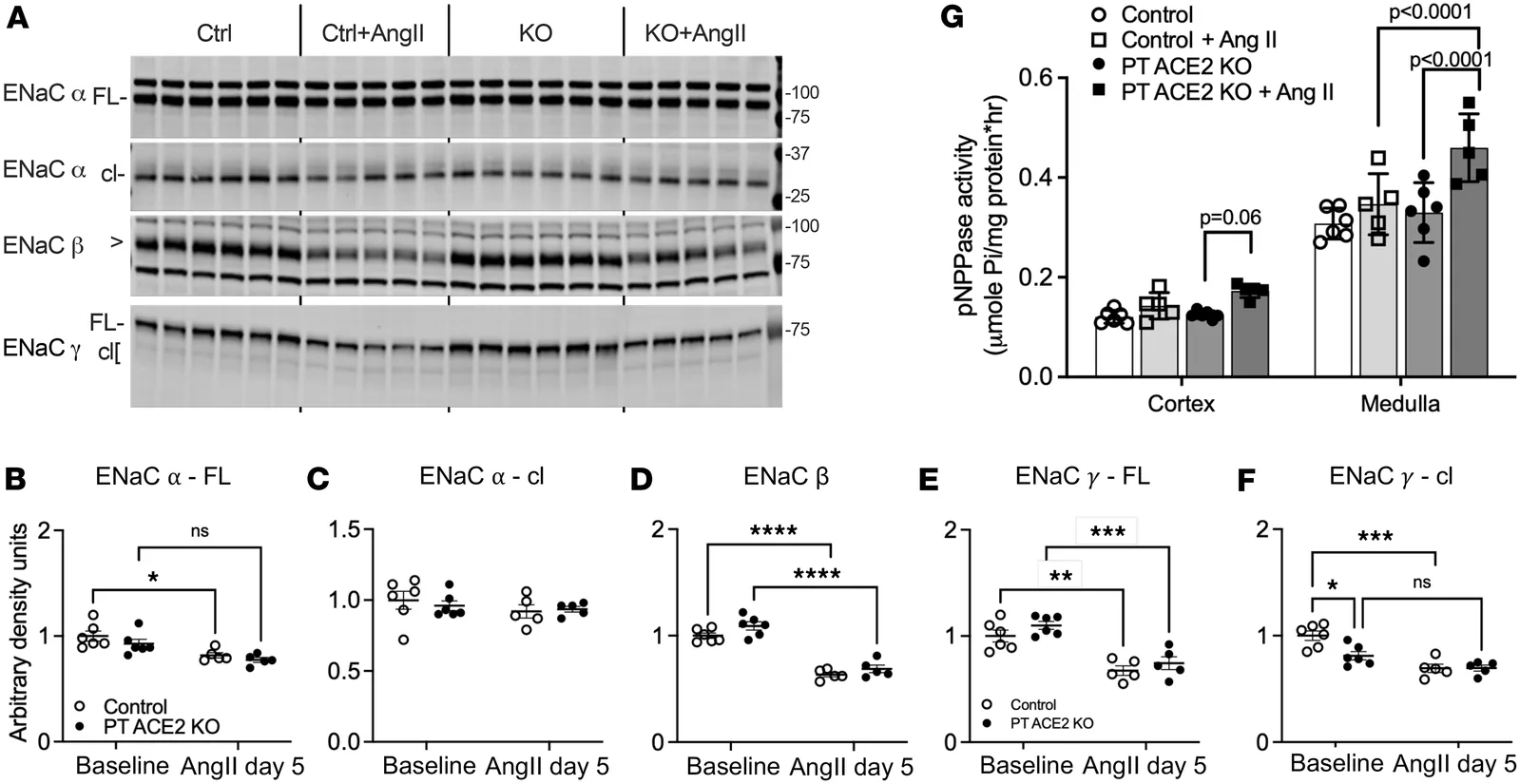
Inappropriate regulation of ENaCγ and Na,K-ATPase activity impairs pressure natriuresis in PT ACE2–KO mice. (**A**) Immunoblots depicting epithelial sodium channel (ENaC) abundance at baseline and with 5 days of AngII. (**B**) Full-length ENaCα (ENaCα - FL) (**C**) Cleaved ENaCα (ENaCα – cl). (**D**) ENaCβ (ENaC β). (**E**) Full-length ENaCγ (ENaCγ-FL). (**F**) Cleaved ENaCγ (ENaCγ-cl). (**G**) K^+^-Dependent p-nitrophenyl phosphatase (pNPPase), a surrogate for Na,K-ATPase activity. Data expressed as mean ± SEM; transporter abundance was analyzed as a 2-way ANOVA with Tukey’s multiple-comparison post hoc analysis. pNPPase activity was analyzed by mixed-effects analysis with Fisher’s LSD pairwise comparison post hoc analysis (*n* = 5–6 mice per group). *P < 0.05, **P < 0.01, ***P < 0.001, ****P < 0.0001.

Intrarenal RAS activity drives increases in sodium reabsorption when effective circulating volume is low and can drive hypertension if RAS activity is too high. In AngII-mediated hypertension, antinatriuretic effects are associated with increased sodium transporter abundance along the cortical thick ascending limb (TAL) to the cortical collecting duct (CCD) (5, 7, 41), which are often balanced by pressure-natriuretic effects in order to decrease sodium transporter abundance in the proximal nephron (41, 42). To determine whether changes in sodium handling support the amplified response to AngII infusion in PT ACE2–KO mice associated with transient increases in kidney AngII levels, we examined abundance of sodium transporters along the renal tubule in Control and PT ACE2 KO at baseline and with 5 days of AngII infusion (Figure 4, A–J, Figure 5, A–J, and Figure 6, A–F). Of the key transporters we profiled at baseline within the PT and medullary TAL, sodium-glucose cotransporter (SGLT2) abundance was higher while the cleaved, activated form of the epithelial sodium channel γ-subunit (ENaCγ) was lower in abundance in PT ACE2–KO mice (Figure 4, A and C, and Figure 6, A and F). More changes in transporter abundance were observed during AngII hypertension. In both Control and PT ACE2–KO mice, AngII infusion decreases the abundance of distal nephron transporters: Na^+^/K^+^/Cl^–^ cotransporter (NKCC2, both total and phosphorylated forms; Figure 5, A–C) and claudin 8 (which prevents sodium back-leak along CCD; Figure 5, A and H). In PT ACE2–KO mice treated with AngII, SGLT2 abundance decreased (Figure 4, A and C), along with a reduction in the phosphorylated form of the aquaporin 2 water channel (AQP2p; Figure 5, A and J) to the same level as Controls. AngII-induced reductions in SGLT2 and AQP2p reflect efforts to maintain homeostasis by enhancing natriuresis and diuresis in PT ACE2–KO mice.

### Inappropriate regulation of ENaC and Na,K-ATPase activity impairs pressure natriuresis in PT ACE2–KO mice.

Notably, we observe a difference between Control and PT ACE2–KO mice with respect to regulation of the ENaC in response to AngII that could contribute to the higher BP after 5 days of AngII infusion (Figure 6, A–F). In Control mice, a 20% reduction in full-length ENaCα (*P* < 0.05) and a 30% reduction in the cleaved, activated form of ENaCγ indicate increased pressure natriuresis (*P* = 0.0001; Figure 6, A, B, and F). Importantly, ENaCα and cleaved ENaCγ were not reduced in response to AngII infusion in PT ACE2–KO mice, suggesting an insufficient natriuretic response to AngII. The inability of PT ACE2–KO mice to regulate ENaC activity in response to AngII may explain the exaggerated BP response observed in PT ACE2–KO mice.

Additionally, we examined Na,K-ATPase activity, a key molecular pump that fuels sodium reabsorption all along the nephron and is the main driver of kidney oxygen consumption. Na,K-ATPase activity was higher in the homogenates from cortex than medulla, as expected (Figure 6G). Na,K-ATPase activity was unchanged by loss of ACE2 from the PT at baseline; however, AngII increased Na,K-ATPase activity in medulla of PT ACE2–KO mice (*P* = 0.0001), with a marginal but nonsignificant increase in cortical activity. In contrast, no increase in Na,K-ATPase was observed in Control animals. Along with differential regulation of ENaC, this finding suggests that the higher local AngII in PT ACE2–KO mice promotes AngII-dependent increases in sodium pump activity, together reflecting a blunted pressure natriuretic response to AngII (Figure 6, A, B, and D).

### Transcriptional changes in the kidney in response to treatment with AngII for 5 days.

**Figure 7:**
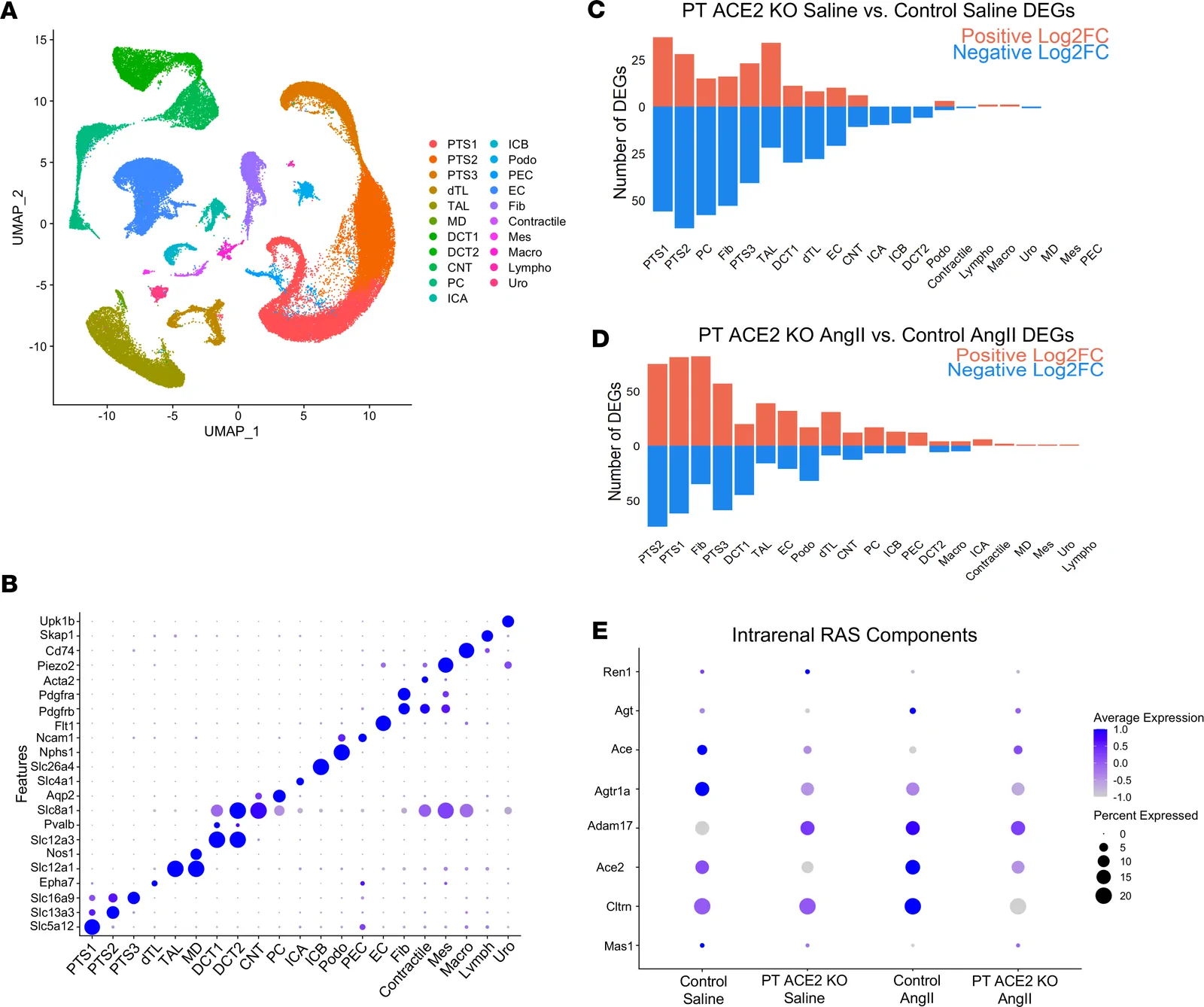
Transcriptional changes in the kidney in response to treatment with AngII for 5 days. (**A**) UMAP plots of all mouse kidney datasets integrated with FindIntegrationAnchors. PTS1; S1 segment of proximal tubule; PTS2, S2 segment of proximal tubule; PTS3, S3 segment of proximal tubule; dTL, descending limb of Loop of Henle; TAL, thick ascending limb of Loop of Henle; MD, macula densa; DCT1, early segment of distal convoluted tubule; DCT2, late segment of distal convoluted tubule; CNT, connecting tubule; PC, principal cells; ICA, type A intercalated cells; ICB, type B intercalated cells; Podo, podocytes; PEC, parietal epithelial cells; EC, endothelial cells; Fib, fibroblasts; Mes, mesangial cells; Macro, macrophages; B & T, B and T lymphocytes; Uro, uroepithelial cells. (**B**) Dot plot displaying gene expression patterns of cluster-enriched markers. (**C** and **D**) Number of differentially expressed genes per cluster in saline- and AngII-treated groups. (**E**) Intrarenal RAS gene expression in proximal tubule segments 1–3, contractile cells, mesangial cells, and macrophages.

To quantify how loss of PT-derived ACE2 affects gene expression in the kidney during periods of hypertension and dysregulated intrarenal RAS, we performed snRNA-seq on cryopreserved kidneys from Control and PT ACE2–KO mice treated with either saline or AngII for 5 days. After quality control filtering, we obtained 80,013 nuclei from 8 mice consisting of 2 replicates for each experimental group (Table 2). We captured all major cell types of the kidney based on canonical kidney cell markers (Figure 7, A and B, and Supplemental Figures 3 and 4) (43). We did not detect any bias in the distribution of cell types based on experimental group (Supplemental Figure 5, A and B).

Next, we quantified the differentially expressed genes (DEGs) for all cell clusters in each group. Comparison of PT ACE2–KO versus Control kidneys with saline or AngII revealed that all 3 segments of the PT were among the clusters with the highest number of DEGs (Figure 7, C and D), demonstrating that loss of ACE2 significantly affects gene expression locally, predominantly within the PT.

Additionally, we took a targeted look at the transcriptomic data by assessing the profile of intrarenal RAS components in multiple cell types in the kidney (clusters: PT segments 1–3, contractile cells, mesangial cells, and macrophages; Figure 7E), which supports an increase in *Ace2* and *Agt* expression in AngII-treated Control kidneys. Increased *Adam17* expression in AngII-treated Control kidneys is consistent with the increased ACE2 cleavage we observed in our urine studies (Figure 7E and Figure 3, C, H, and I). In Control mice, AngII treatment also induced an increase in *Cltrn* (collectrin), a homologue of the ACE2 membrane-anchoring domain, an effect that was not seen in PT ACE2–KO mice. In PT ACE2 KO kidneys, *Ace*, *Agt*, and *Adam17* expression increased with AngII treatment, consistent with activation of the intrarenal RAS.

### Differential pathway enrichment in PT ACE2–KO and Control mice.

**Figure 8:**
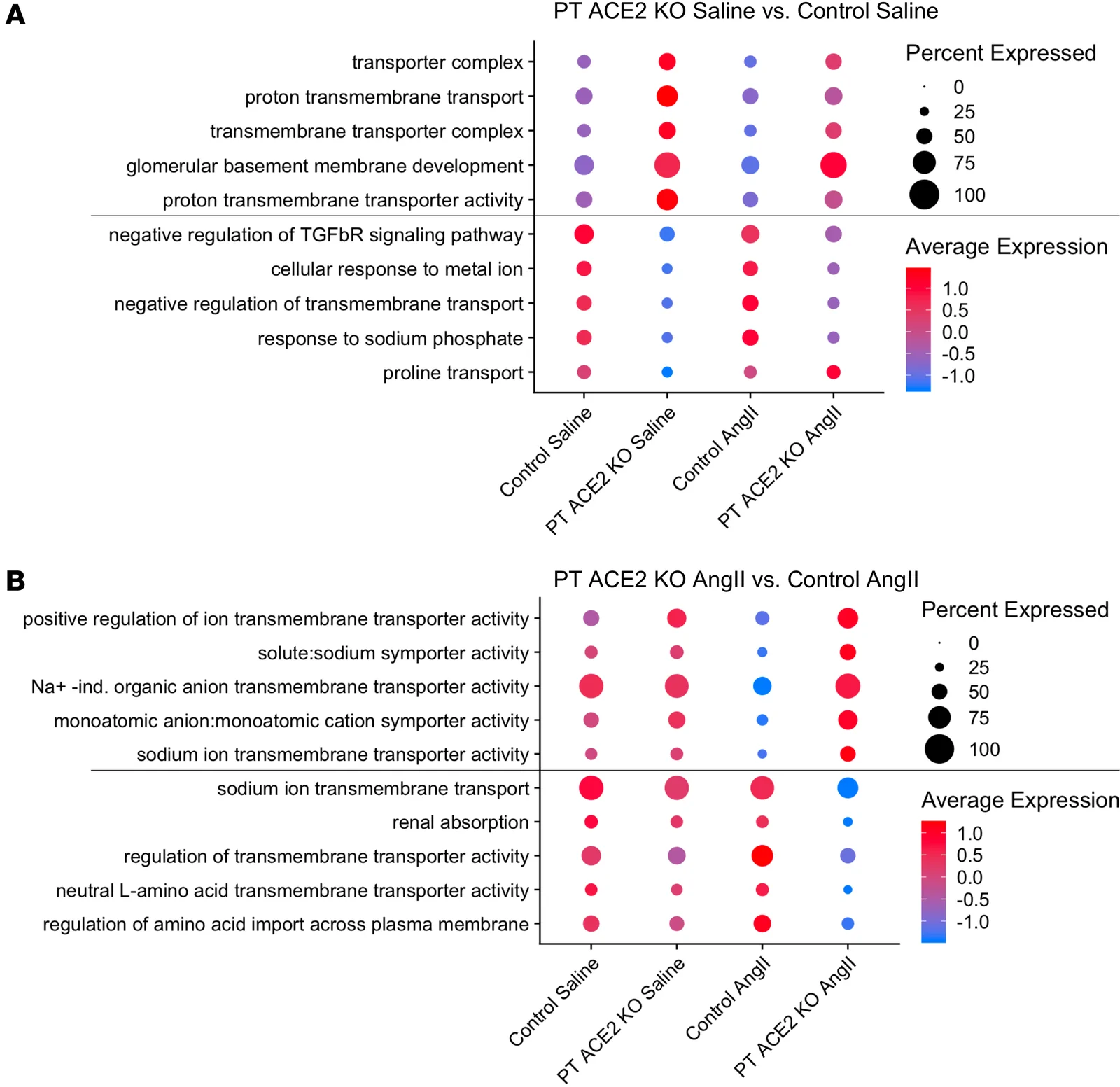
Differential pathway enrichment in PT ACE2–KO and Control mice. (**A**) Enrichment of pathways found significant in PT ACE2 KO Saline versus Control Saline. (**B**) Enrichment of pathways found significant in PT ACE2 KO AngII versus Control AngII.

To further explore changes in gene expression in the PT, we employed gene set enrichment analysis (GSEA) using an a priori defined set of genes associated with known biological functions, and we compared pathways unique to either saline- or AngII-treated PT ACE2–KO kidneys relative to their Control counterparts. In saline-treated PT ACE2–KO mice, pathway analysis demonstrated more positive enrichment of well-known PT-related functions, including upregulation of transporter complex (*q* = 0.032), proton transmembrane transport (*q* = 8.18 × 10^–5^), proton transmembrane transporter activity (*q* = 2.20 × 10^–6^), and glomerular basement membrane development (*q* = 0.035) compared with saline-treated Control kidneys (Figure 8A and Supplemental Table 4). Concomitant downregulation of transcripts related to regulation of transforming growth factor β receptor (TGFβR) signaling (*q* = 0.026), cellular response to metal ion (*q* = 0.012), and negative regulation of transmembrane transport (*q* = 0.029) were also seen in saline-treated PT ACE2–KO mice, along with downregulation of response to sodium phosphate and proline transport. Interestingly, genotype-dependent transcriptional differences observed under baseline conditions were preserved following AngII treatment, underscoring the strong regulatory influence of ACE2 on PT function.

However, AngII treatment induced transcriptional differences between Control and PT ACE2–KO mice that were absent under baseline conditions (Figure 8B and Supplemental Table 4). Specifically, in Control mice, AngII treatment was associated with negative enrichment of ion and solute transport–associated genes, including solute:sodium symporter activity (*q* = 2.16 × 10^–5^), sodium-independent organic anion transmembrane transporter activity (*q* = 0.002), and positive regulation of ion transmembrane transporter activity (*q* = 0.039), while in PT ACE2 KO kidneys, these pathways were enriched similar to saline-treated Control and PT ACE2–KO groups. Assessment of pathways associated with core PT reabsorption processes reveal the opposite pattern: AngII-treated PT ACE2–KO kidneys demonstrate lower expression of renal absorption (*q* = 0.003), sodium ion transmembrane transport (*q* = 0.003), and regulation of amino acid import across the plasma membrane (*q* = 0.026), while in the Control AngII group, enrichment of these pathways is maintained at levels similar to the saline-treated Control and PT ACE2–KO groups. Together, these findings demonstrate that loss of ACE2 in the PT fundamentally reshapes the transcriptional response to AngII, particularly across key transport pathways.

Loss of ACE2 from the PT led to significant transcriptional changes of apical membrane-related genes (Supplemental Figure 6A and Supplemental Table 5). Compared with the saline-treated Control group, saline-treated PT ACE2–KO mice displayed significant negative enrichment of pathways involved in regulation of actin filament organization (*q* = 0.0088), regulation of actin cytoskeleton organization (*q* = 0.02), cluster of actin-based cell projections (*q* = 0.0004), cell projection membrane (*q* = 0.033), apical plasma membrane (*q* = 8.32 × 10^–6^), and brush border (*q* = 0.010). Treatment with AngII led to greater dissonance between PT ACE2–KO and Control gene expression; genes associated with these pathways were further downregulated in PT ACE2 KO AngII kidneys and upregulated in Control AngII samples — e.g., brush border (*q* = 3.82 × 10^–16^), cell projection membrane (*q* = 1.54 × 10^–14^) and cluster of actin-based projections (*q* = 1.23 × 10^–13^). These changes reveal categorical differences of PT response to AngII in the absence of PT ACE2.

To complement our analysis of transporter protein abundance (Figure 4, Figure 5, and Figure 6), we also examined transporter gene expression along the nephron (Supplemental Figure 7). Assessment of transporters along the more proximal nephron (clusters: PT segments 1-3 and the TAL revealed that AngII led to contrasting expression of *Cldn10*, *Aqp1*, and *Cldn2* between PT ACE2–KO and Control groups (Supplemental Figure 7A). Even greater discordance in transporter gene expression between AngII-treated PT ACE2–KO and Control groups was seen along the distal nephron segments (TAL, macula densa, distal convoluted tubule 1 and 2, connecting tubule, A and B type intercalated cells, and urothelium clusters; Supplemental Figure 7B), including upregulation of *Scnn1g*, *Scnn1a*, *Slc12a3*, and *Aqp2*. Importantly, differential transcriptional regulation of ENaC subunits and RAS components mirror the changes in transporter abundance and levels of RAS components measured by immunoblotting and enzymatic assay, and they suggest exaggerated AngII effects in PT ACE2–KO mice.

## Discussion

Our study establishes a critical role for PT-derived ACE2 in hypertension pathogenesis and highlights mechanisms by which ACE2 in the PT attenuates hypertension. Indeed, our findings indicate that ACE2 in the PT is a major regulator of the intrarenal RAS through its actions to metabolize AngII and abrogate its tubular actions. In Control mice, increases in renal *Ace2* expression, abundance, and activity signify an early adaptive response to AngII-induced hypertension, and the absence of these actions in the PT ACE2–KO mice results in exaggerated hypertension. This effect of PT-derived ACE2 to protect against hypertension is most readily apparent during the first week of AngII treatment, mitigating deleterious intrarenal RAS activation, preventing overstimulation of sodium reabsorption, and reducing cardiac hypertrophy.

PT ACE2–KO mice demonstrate an exaggerated BP response to AngII infusion during the first week of treatment (Figure 3A), followed by recovery to Control MAP levels during the second week. The enhancement of hypertension in PT ACE2–KO mice is limited compared with global ACE2 deletion, where we found an exaggerated hypertensive response throughout the entire period of chronic AngII infusion (39). In contrast to the BP response to AngII in global ACE2-KO mice, the availability of ACE2 in extrarenal tissues of PT ACE2–KO mice may allow for systemic ACE2 to reach the kidney to defend against elevated AngII levels. Nonetheless, the enhancement of hypertension in PT ACE2 KO causes more severe end-organ damage compared with Control animals, reflected by increased HW/TL and increased LV mass determined by echocardiography, as well as increased albuminuria. We observe that hypertrophic remodeling occurs rapidly in response to high-dose AngII treatment (139.9 ± 14.4 mmHg in Control versus 148.7 ± 17.5 mmHg in PT ACE2 KO, *P* = 0.0007 at 5 days of AngII), similar to the rapid hypertrophic growth seen in response to the transverse aortic constriction (TAC) surgical model of pressure overload. In the TAC model, increases in HW/BW occur as early as 2–7 days (44, 45). Adding to the confidence in our data, the HW/TL data were aggregated from multiple independent experiments and, thus, represent a consistent hypertrophic response to AngII in the PT ACE2–KO mice. The increase in hypertrophic cardiac remodeling in the PT ACE2 KOs suggests that even a transient rise in afterload pressure and accompanying elevation in renal AngII levels are sufficient to induce pathological hypertrophic remodeling (34). The disruption of normal renal processes, which results from loss of PT ACE2, may signify a predisposition to renal-cardio syndrome.

Increased BP response to AngII infusion in PT ACE2–KO mice is accompanied by intrarenal accumulation of AngII and a trend toward increased expression of other RAS components (angiotensinogen), without concomitant change in urinary ACE2 levels. By contrast, in the Control mice, urinary ACE2 abundance and activity increased significantly during the initial days of AngII infusion. This response in Control mice reflects an early induction of ACE2 activity in the PT, accompanied by shedding of sACE2 into the urine. The increased renal and urinary ACE2 in Control mice may affect BP elevation in 2 ways, (a) mitigation of intrarenal accumulation of AngII and activation of kidney RAS, and (b) generation of Ang(1–7), together dampening the BP elevation. However, during AngII infusion Control mice demonstrate downregulation of the Ang(1–7) receptor, *Mas1*, whereas no change in *Mas1* expression is observed in PT ACE2–KO mice. This suggests that the reduced sensitivity to AngII infusion observed in Control mice is primarily due to mitigation of AngII levels rather than Ang(1–7)/Mas receptor activity. Intrarenal RAS activation has previously been demonstrated to promote hypertension (46), but this is the first report to our knowledge of direct involvement by ACE2 as a modulator. Finally, as components of the systemic RAS were not significantly altered in the PT ACE2 KOs (Supplemental Figure 1), PT ACE2 activity represents a renal-focused mechanism for BP regulation.

ACE2 can be cleaved from the membrane, resulting in a smaller, catalytically active ACE2 fragment (sACE2), which can translocate from the cell of origin. Using a kidney cross-transplantation model, we previously demonstrated that urinary sACE2 arises from renal and systemic sources, and we demonstrated that sACE2 originating outside the kidney is filtered into the urine (27) and is detectable on urine immunoblots. The current study confirms this observation, while further demonstrating that the PT is the predominant source of urinary ACE2. While the source of the 98 kDa form of ACE2 cannot be definitively determined in our studies, the molecular size is consistent with the full-length protein and may reflect intact ACE2 protein contained in sloughed cells or present in urinary extracellular vesicles (47, 48). Nonetheless, our studies clearly indicate that the PT is the main source of full-length ACE2 in urine as it is virtually absent in PT ACE2–KO mice. In Control urine, excess sACE2 present at 6 days is likely due in part to proteolytic processing of PT-derived ACE2 within the urinary space (49). Alternatively, the smaller ACE2 fragment may indicate filtration of systemically derived sACE2 or proteolytic processing of non-PT–derived ACE2 within the tubular lumen (27), either of which may account for the low levels of sACE2 in the urine of PT ACE2–KO mice. Impaired metabolism of AngII in PT ACE2 KO kidneys may induce ADAM17 activity (50), as suggested by increased *Adam17* RNA expression in both Control and PT ACE2–KO mice treated with AngII, thus promoting cleavage of ACE2 from either systemic or non-PT renal sources and resulting in the urinary sACE2 levels detectable during the first week of AngII infusion.

To identify the molecular mechanisms resulting from elevated AngII levels and intrarenal RAS activation, we measured renal transporter abundance in Control and PT ACE2–KO mice. At baseline, loss of PT-derived ACE2 was associated with few changes, mainly an increased abundance of SGLT2 (Figure 4, A and C) and reduced cleaved ENaCγ (Figure 6, A and F), with lower expression of *Slc5a2* and *Scnn1g* (Supplemental Figure 7B). Differences in SGLT2 abundance and cleaved ENaCγ at baseline may account for the lack of a BP difference between Control and PT ACE2–KO mice. Together, these results indicate that proximal tubular ACE2 has modest effects on transporter abundance necessary to maintain sodium homeostasis at baseline.

On the other hand, AngII-mediated hypertension induces differential alterations in transporter abundance in Control and PT ACE2–KO mice to enhance natriuresis and diuresis in support of extracellular fluid volume homeostasis. In both animal groups, we observed decreased abundance of total and phosphorylated NKCC2, Claudin 8, ENaCβ, and full-length ENaCγ (Figure 5, A–C, and H, and Figure 6, A, D, and E). *Slc12a1* and *Cldn8* RNA expression were increased in Control kidneys and suppressed in PT ACE2–KO kidneys, despite similarities in protein abundance, perhaps reflecting differences in post-translational regulation (Supplemental Figure 7B). Importantly, Control mice alone exhibited a reduction in cleaved (activated) ENaCγ (Figure 6, A and F), which supports final renal adjustments to sodium excretion and BP regulation (51, 52). Increased Na,K-ATPase activity in PT ACE2–KO mice supports aberrant ENaCγ activity in PT ACE2–KO mice by maintaining sodium and potassium concentration gradients. In the absence of appropriate ENaCγ activity downregulation, PT ACE2–KO mice uniquely demonstrated a reduction in SGLT2 and phosphorylated AQP2 in response to AngII (Figure 4, A and C, and Figure 5, A and J), representative of alternative means of compensating for overstimulation of sodium reabsorption. While reductions in SGLT2 and AQP2p abundance in PT ACE2–KO mice serve to maintain sodium balance during AngII treatment, these natriuretic and diuretic adjustments are ultimately insufficient to overcome persistent activation of ENaCγ, allowing BP to rise during this critical window of early AngII stress.

Previously, we demonstrated that loss of the type 1A angiotensin receptor (AT1AR) from the PT impaired the antinatriuretic response to chronic infusion of AngII, leading to lower BP relative to control mice. Here we see the inverse effect of AngII in the absence of PT ACE2, in which aberrant ENaCγ activity and concomitant downregulation of SGLT2 and AQP2p together shift the pressure natriuretic curve to the right, increasing BP. These results are in line with earlier studies that implicate dysregulation of ENaCγ cleavage in hypertension (52, 53). Dysregulation of ENaCγ activity in PT ACE2–KO mice may occur due to inflammation resulting from increased intrarenal AngII accumulation, as has been previously shown (52).

In addition to differential regulation of transporter abundance, PT ACE2–KO and Control mice display distinct transcriptional pathway enrichment in response to AngII (Figure 8, A and B). Control mice appear to downregulate genes involved in ion transporter activity, whereas PT ACE2–KO mice downregulate genes associated with amino acid reabsorption. ACE2 has been associated with amino acid transport within the intestinal tract, as expression of the Hartnup sodium-dependent neutral amino acid transporter, B^0^AT1, is dependent on ACE2 expression, whereas B^0^AT1 expression and activity along the PT brush border is dependent on the ACE2 homolog, collectrin (54). Consistent with previous work that demonstrated that collectrin expression was protective against early hypertension (55), Control mice in our study exhibited lower MAP and increased *Cltrn* RNA expression after 5 days of AngII, while PT ACE2–KO mice displayed reduced *Cltrn* expression with AngII (Figure 7E). No differences in collectrin mRNA expression were observed between PT ACE2–KO and Control mice at baseline, suggesting that deletion of ACE2 from the PT does not by itself affect collectrin expression. Nonetheless, it is possible that AngII-dependent alterations in collectrin expression affect amino acid reabsorption and brush border function, as seen in PT ACE2–KO animals. Interestingly, we observed a significant effect of ACE2 loss on PT brush border function and structure by examination of differential gene expression in pathways involving the apical plasma membrane and actin organization (Supplemental Figure 6A). Widespread gene expression changes resulting from the loss of PT ACE2 suggests that ACE2 is integral in brush border structural integrity. Genes associated with these pathways were further suppressed in PT ACE2–KO mice after AngII treatment, opposite to the effect seen in Control kidneys, suggesting that loss of ACE2 depresses the normal brush border response to AngII. While not the focus of our current study, the role of ACE2 in maintaining PT brush border may offer additional insight into fluid and solute handling by the PT under control of the intrarenal RAS and may offer important insights into SARS-CoV-2 infectivity.

Together, the results of this study demonstrate that PT-derived ACE2 plays an essential role in protecting against the early phase of AngII hypertension and end-organ damage, and it is a key factor in mediating intrarenal RAS activity in hypertension. Our study identifies the PT as a major source of sACE2 in urine, which likely contributes to AngII breakdown and attenuation of intrarenal RAS activation. PT ACE2 is necessary for appropriate regulation of ENaCγ activity, and reduced cleaved ENaCγ at baseline may limit sodium avidity and protect against elevated BPs. Importantly, since loss of ACE2 from the PT was sufficient to cause increased hypertension and cardiac hypertrophy without concomitant alterations in systemic RAS, our work further supports a renal origin for RAS-driven cardiac hypertrophy (34). ACE2 shed from the PT may facilitate the degradation of AngII along the lumen of the nephron and affect transporter abundance downstream, as suggested by both protein abundance and snRNA-seq data, thus affecting disease processes where upregulation of intrarenal RAS is injurious. Our findings support targeting the PT using regulators of ACE2 processing or recombinant sACE2 protein as an important strategy in treating hypertension and other kidney diseases.

## Methods

### Sex as a biological variable.

Only male mice were studied as both targeted genes are on the X chromosome. It is unknown whether the findings are relevant for female mice.

### Experimental animals.

Mice with PT specific deletion of ACE2 (PT ACE2 KO) were generated by crossing 129/SvEv *Ace2*^fl/y^ mice developed by our lab (36) to 129/SvEv *Pepck-Cre* mice (36), with each line back-crossed for > 6 generations to obtain fully inbred 129/SvEv ACE2^–/y^ PT ACE2–KO mice. PT ACE2–KO mice were compared with age-matched littermate Controls that carried the floxed *Ace2* allele but no *Cre*. The generation of global ACE2-KO mice has been previously described (39). Mice aged 8–26 weeks (average 15.4 ± 4.4 weeks) were used for this study.

### Measurement of BP.

BP was measured in conscious, unrestrained mice using a radiotelemetry system previously described (5, 27). Mice were allowed to recover from surgery and regain normal circadian rhythm for 1 week prior to experimental BP recording. Throughout the measurement period, mice were housed in individual cages in a quiet monitoring room. BPs were measured continuously over a 10-second interval every 5 minutes, and recordings were collected, stored, and analyzed using Dataquest ART software (version 4.1; Transoma Medical). Baseline BPs were measured continuously for 5 days. AngII peptide (Sigma-Aldrich A9525) was continuously infused for 2 weeks using subcutaneous osmotic minipumps (Alzet 1004) preloaded with AngII dissolved in sterile 0.9% sodium chloride for an infusion dose of 1,000 ng kg^–1^ min^–1^ (5, 39). MAP, heart rate, and systolic and diastolic BP values were averaged for the baseline and AngII infusion period. Animals were housed in individual metabolic cages for 24-hour urine collection (Hatteras Instruments). Throughout the study period, animals had free access to normal chow diet and water. At the end of the experiment, animals were euthanized under 4.5% isoflurane anesthesia by exsanguination, and plasma, kidneys, and hearts were collected. MAP data (*n* = 16) were analyzed by 2-way repeated-measures ANOVA with Tukey’s post hoc analysis using GraphPad software.

### Cardiac phenotyping.

Heart weight to tibia length (HW/TL) data were aggregated from multiple independent experiments (including saline challenge and sodium balance studies). Tibia length was measured using digital calipers. To assess LV wall thickness in vivo, echocardiography was performed using a Vevo 2100 with a ms400 transducer at 38MHz (FUJIFILM VisualSonics). Mice were sedated with inhaled isoflurane (1.5%–2.0%) and core temperature maintained at 37°C. Images were obtained in the parasternal long-axis plane and the midpapillary short-axis plane. Image registration to ensure similar imaging planes was based on anatomic features (papillary muscles and trabecular features). The same measures were obtained for epicardial area and major axis length. Left ventricle wall thickness was obtained by subtracting the average LV endocardial end diastolic radius from the average LV epicardial end diastolic radius. Measurements were taken at baseline, and with AngII infusion on days 7 and 14.

### ACE2 protein and enzymatic activity assessment.

ACE2 activity was determined as described previously (19) following incubation with the intramolecularly quenched synthetic ACE2-specific substrate Mca-APK-Dnp (Anaspec) (19). For immunoblots, kidney homogenates were prepared as previously described, and concentration was determined by Bradford assay (Bio-Rad Protein Assay) (27). In total, 50 μg kidney homogenates were loaded into a 4%–12% polyacrylamide gel. For urine samples, equal volumes of undiluted urine were loaded into a 4%–12% polyacrylamide gel. Immunoblots were run using a previously established protocol (39), and ACE2 was detected 1:1,000 anti-ACE2 primary antibody (Abcam ab108252) followed by 1:5,000 dilution anti-Rabbit horseradish peroxidase conjugated secondary antibody (Thermo Fisher Scientific, 31460). Super Signal West Pico chemiluminescence kit (Thermo Fisher Scientific) was used to develop immunoblots with exposure to Kodak RX film. Coomassie stain was performed as a protein loading control in the same membranes without stripping membrane.

### RNA isolation and analysis.

Relative levels of mRNA for *Ace2* were measured in kidney cortices from each group. RNA was isolated and reverse transcription performed using qScript cDNA Supermix (Quanta Biosciences). qPCR was carried out using the SYBR Green PCR Master Mix (Applied Biosystems). The amount of target gene relative to endogenous control was determined by the ΔΔCT method. The following primer sequences were used: *Ace2* forward, 5′-ACTCACAGCAACCCTCCAAG-3′; *Ace2* reverse, 5′-ATCACCACCAAGCTGTTTCC-3′ (Amplicon size: 236bp); *Gapdh* forward, 5′-TCACCACCATGGAGAAGGC-3′; and *Gapdh* reverse, 5′-GCTAAGCAGTTGGTGGTGCA-3′ (Amplicon size: 168bp).

Gene expression was assessed using TaqMan assays for renin (Mm02342887_mH) and 18S rRNA (Mm03928990_g1) (Applied Biosystems). Reactions were performed using TaqMan Fast Advanced Master Mix (Applied Biosystems, 4444556) according to the manufacturer’s instructions.

### Tissue handling and immunofluorescence.

Kidneys were gravity perfused via retrograde abdominal aortic perfusion with PBS with heparin for 3 minutes, followed by 4% paraformaldehyde (PFA) in PBS for 5 minutes. Kidneys were fixed in 4% PFA, cryoprotected in 30% sucrose overnight, and frozen in optimal cutting temperature (OCT) compound mounting medium for sectioning, and 10 μm transverse sections were cut on a cryostat. To reduce fixative-induced autofluorescence, sections were rinsed in 10 mg/mL sodium borohydrate 3 × 10 minutes and rinsed in PBS 3 × 5 minutes. Sections were then incubated with blocking buffer (5% BSA, 2.5% donkey serum, 0.1% Triton X-100) in PBS for 1 hour, incubated with goat anti-ACE2 (1:200, Abcam ab108252) antibody overnight. After rinsing with PBS, slides were incubated with Alexa Fluor 568–conjugated goat IgG-specific antibody and Fluorescein Lotus Lectin (LTL, 1:200, Vector Laboratories FL-1321), rinsed, cover-slipped in Prolong Gold Mounting Media, and visualized by fluorescence microscopy on a Keyence epifluorescence microscope.

### AngII peptide measurements.

Renal AngII peptide levels were determined with an enzyme immunoassay (ELISA kit S-1133, BMA Biomedicals,). For this, one-half of a kidney was weighed and homogenized in ice-cold methanol. After centrifugation at 12,000*g* for 10 minutes at 4°C, the collected supernatant was dried by centrifugal evaporation. Dried pellets were rehydrated with the buffer provided by the kit. AngII was measured following manufacturer’s instruction as previously described (51) and expressed as femtomole per gram of wet kidney.

### Homogenate preparation and immunoblot analysis of transporter abundance.

Homogenate preparation and immunoblotting protocols were as described in detail recently (6). In brief, kidneys were defrosted, and cortex and medulla were dissected on ice before being homogenized in isolation buffer containing protease and phosphatase inhibitors using an Ultra-Turrax T25 (IKA Labortechnik). Centrifugation was performed to isolate a 2,000*g* supernatant (“homogenate”); single-use aliquots were quick frozen and stored at –80°C. For semiquantitative immunoblots, protein concentrations were determined using the BCA assay (Pierce, Thermo Fisher Scientific), and equivalent protein loading per sample per lane was validated in Coomassie stained gels, as described previously (6, 56); the gel-loading was judged equivalent if variability between samples was less than 10%. Samples were assessed at full and half amounts (Supplemental Table 1), electrophoretically blotted onto a single piece of PVDF membrane (Immobilon FL; EMD Millipore), and processed with primary and secondary antibodies (Supplemental Table 1). Signals were detected with the Odyssey Infrared Imaging System (LI-COR) and quantified by accompanying software. Arbitrary density units were normalized to the mean intensity of the Control group, defined as 1.0. Since each sample was assayed twice (full and half amounts), mean values were compiled for statistical analysis by 2-way ANOVA using Graph Pad software.

### K^+^-Dependent p-nitrophenyl phosphatase (K-pNPPase) enzymatic assay.

K^+^-pNPPase activity was measured in kidney cortical and medullary homogenates as classically described (57). K^+^-pNPPase measures K- dependent ATPase activity, a surrogate for Na,K-ATPase activity in kidney where H,K-ATPase activity is far lower (58). Results are expressed as μmole inorganic phosphate produced/mg protein/assay time.

### snRNA-seq and transcriptional analysis.

Nuclear isolation and snRNA-seq analyses were modified from those recently described by Su et al. (59). See Supplemental Methods for a detailed description of nuclear isolation protocol and snRNA-seq analysis approach.

### Quantification of systemic RAS.

EDTA plasma was collected by cardiac puncture at the time of tissue harvest and flashed frozen and stored at –80°C until analysis. Levels of angiotensin peptides: Ang(1–10), Ang(1–9), Ang(1–8), Ang(1–7), Ang(2–8), Ang(3–7), Ang(3–8), and Ang(1–5) were quantified using a validated targeted liquid chromatography–mass spectrometry (LC-MS) assay, performed at the University of Southern California Mann School of Pharmacy with a dynamic range from 10 pg/mL to 100 ng/mL, where the lower limit of quantification (LLQ) of 10 pg/mL. Urinary Aldosterone concentrations were measured with an Aldosterone ELISA kit (Cayman Chemical, 501090). Samples were processed and analyzed according to the manufacturer’s instructions, and concentrations were calculated from a standard curve.

### Sodium balance study.

As described previously (34) animals were singly housed in metabolic cages and fed a gel diet (10 g/day) containing water, nutrients, 457 mg/kg sodium, and 1.4 g/kg potassium (Nutra-Gel Diet #S5769, BioServ). After 3 days of baseline urine collection, mice were implanted with AngII-osmotic minipumps as described above and immediately returned to metabolic cages for 5 days. Daily urine volume, food weight, and animal weight were recorded. Urinary sodium and potassium were determined using Flame Photometry. Sodium/potassium intake and output were calculated using food weight or urine volume, respectively. Separately, mice were subjected to saline challenge test under baseline and AngII conditions as previously described (60). Plasma and urine CCr were determined by capillary electrophoresis at the University of Texas at Southwestern.

### Assessment of renal injury.

Terminal blood draw via cardiac puncture was used to assess serum blood chemistries at experimental endpoints. Measurement of blood urea nitrogen and serum creatinine was performed using an iSTAT analyzer (Abbott Laboratories). The albumin/creatinine ratio (ACR) was calculated from urinary albumin and creatinine measurements. Albumin was quantified using the Albuwell M ELISA kit (Ethos Biosciences, 1011), and creatinine was measured using the Creatinine Companion kit (Ethos Biosciences, 1012), following the manufacturers’ instructions.

### Statistics.

Statistical analysis was performed using GraphPad Prism version 10.0.3. Values for each parameter are expressed as the mean ± SEM with individual data points plotted when possible. Transporter abundance was analyzed as a 2-way ANOVA with Tukey’s multiple-comparison post hoc analysis (GraphPad Prism). Urinary ACE2 levels were analyzed by an unpaired 2-tailed *t* test with Welch’s correction. The statistical analysis used for each comparison is described within each figure legend. Statistical significance was defined as a *P* value less than 0.05. 

### Study approval.

All animals were bred, housed, and maintained in an Association for Assessment and Accreditation of Laboratory Animal Care International-accredited animal facility at the Durham Veterans Affairs Health Care System or Oregon Health and Science University, in accordance with the *Guide for the Care and Use of Laboratory Animals* (National Academies Press, 2011). Approval for animal care and experiments was granted by Institutional Animal Care and Use Committees at all institutions.

### Data availability.

Values for all data points are included in the Supporting Data Values file. All snRNA-seq data (FASTQ, Cell Ranger Outputs, and Seurat Object) have been uploaded to GEO (GSE253448, reviewer token: gzepkmgabhmhvqp) and bioinformatic code used for analysis and creation of figures has been published on github (https://github.com/JonathanWNelson; commit ID 81d21bd). Additionally, the dataset is hosted online as a ShinyCell app (61) (https://nelsonlab.shinyapps.io/PTACE2KO_Kidney/) to allow exploration of the dataset through an online browser.

## Author contributions

ZX, DIOM, TMC, and SBG designed the study. JME, ZX, DIOM, NM, JFG, DLL, DLR, HHH, JWN, MWH, NK, BC, and SBG carried out experiments. JWN, DIOM, ZX, JME, DLR, AAM, DLL, BC, SL, and SBG analyzed the data. JME, JWN, DLR, HHH, and SBG made the figures. JME, JWN, DIOM, TMC, and SBG drafted the paper. All authors approved the final version of the manuscript.

## Conflict of interest

The authors have declared that no conflict of interest exists.

## Funding support

This work is the result of NIH funding, in whole or in part, and is subject to the NIH Public Access Policy. Through acceptance of this federal funding, the NIH has been given a right to make the work publicly available in PubMed Central.

- 2009 Alaska Kidney Foundation – ASN Research Grant (SBG).
- R01 DK098382 (SBG).
- P30 DK096493 (SBG).
- 5T32 DK00773 (DIOM).
- AHA Beginning Grant in Aid (SBG).
- NIH K01DK121737 (JWN).
- AHA 20CDA35320169 (JWN).
- The Collins Medical Trust (JWN).
- NIH 5T32GM109835-03 (JME).
- NIH 5TL1TR002371-04 (JME).
- AHA Predoctoral Fellowship 916639 (JME).
- ASN Predoctoral Fellowship (JME).

## Supplementary Material

Supplemental data

Supplemental data set 1

Supplemental data set 2

Supplemental data set 3

Supporting data values

## Figures and Tables

**Table 1:**
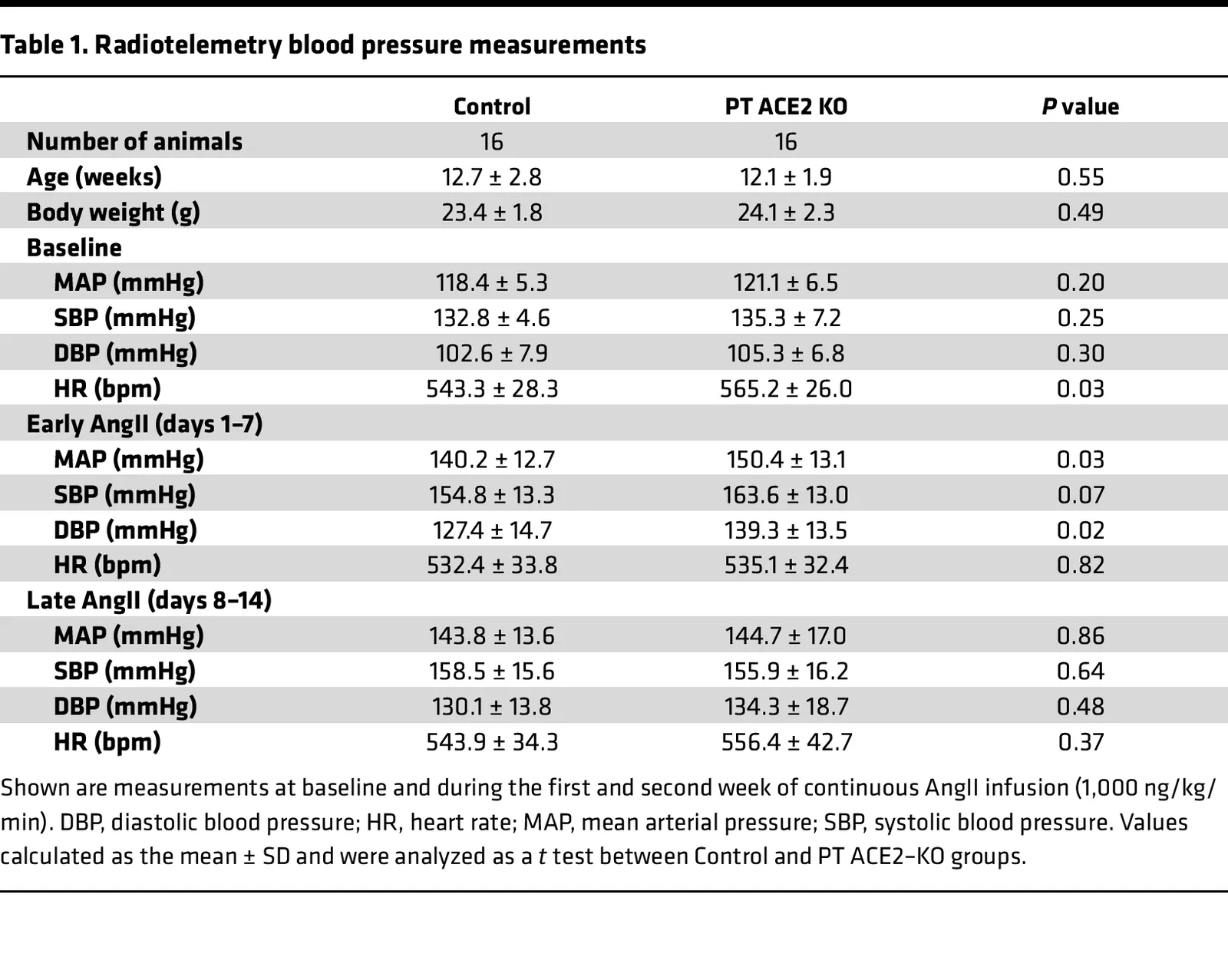
Radiotelemetry blood pressure measurements

**Table 2:**
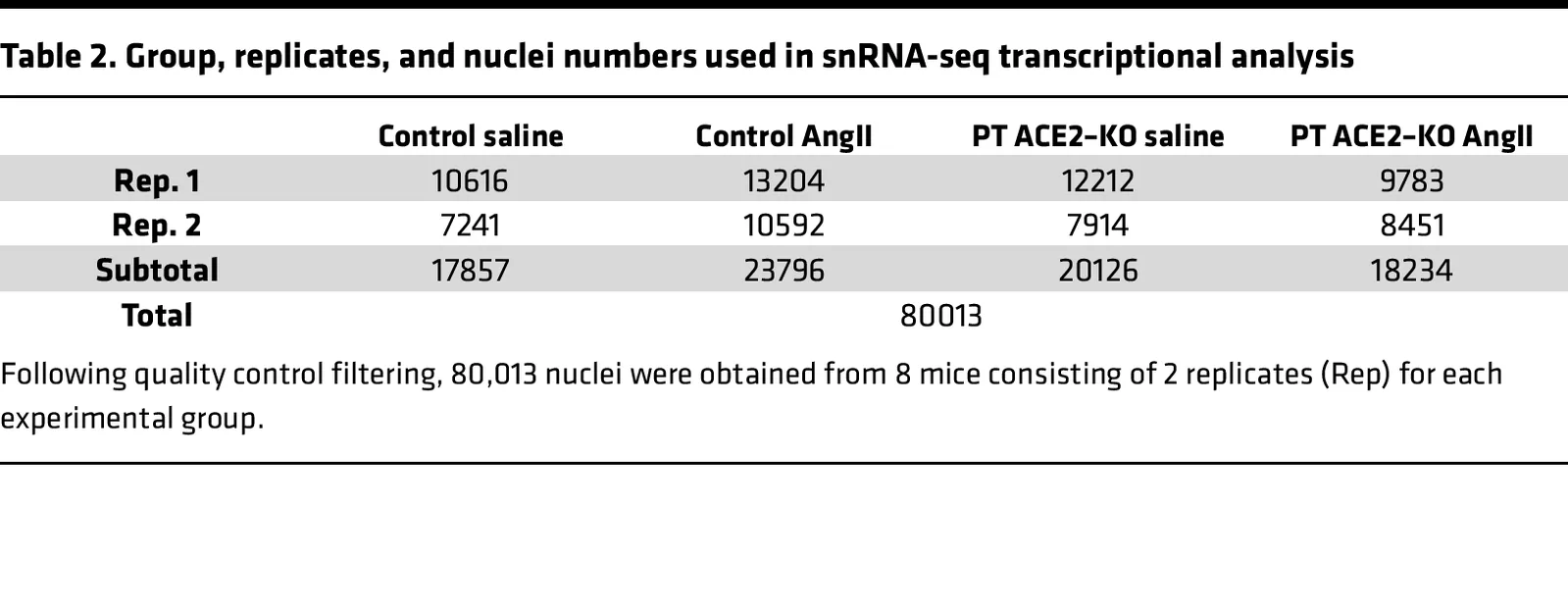
Group, replicates, and nuclei numbers used in snRNA-seq transcriptional analysis

## Version 1 09/22/2026

Electronic publication
