# Supplementary material for: Proximal tubule ACE2 mediates early responses to hypertension by regulating intrarenal RAS and sodium homeostasis
Source: JCI Insight. 2026 Sep 22;11(18):e179960. doi: 10.1172/jci.insight.179960 (PMC13596715; doi:10.1172/jci.insight.179960)
Supplement: Supplementary file 1 [file jciinsight-11-179960-s089.pdf]

Supplemental methods

Nuclear isolation and single nuclear RNA sequencing (snRNAseq) analyses were modified from those recently described by Su et al (1).

Nuclei isolation from mouse kidney and fluorescence-activated nuclei sorting (FANS). Kidneys were removed after PBS washout via aortic perfusion and then snap-frozen in liquid nitrogen. Frozen tissue was stored at -80 °C until subsequent tissue processing. We slightly modified the TST (INNER cell) nuclei extraction (2) and the kidney nuclei isolation protocol (3,4). The nuclei isolation buffer (NIB) contains 146 mM NaCl, 10 mM Tris-HCl (pH 7.5), 1 mM $CaCl_2$, 21 mM $MgCl_2$, 0.03% Tween-20, 0.01% BSA, and 1 tablet of cOmplete ULTRA per 10 ml NIB. The samples were ground for 30 times in 2ml NIB1 (4mL NIB + 20µl RNasin Plus + 20µl SUPERaseIN) with a 2ml Dounce grinder and a loose pestle, then homogenate was passed through a 200 µm strainer. The homogenate was ground for 15 times with a tight pestle, then 2 ml NIB1 was added, and the homogenate was incubated for 5 min on ice. The homogenate was passed through a 40 µm strainer, then centrifuged at 500g for 5 min at 4°C. The pellet was resuspended in 4 ml NIB2 (4mL NIB + 4µl RNasin Plus + 4µl SUPERaseIN) and the suspension was incubated on ice for 5 min, then centrifuged at 500g for 5 min at 4°C. The pellet was resuspended in 1.5 ml of nuclei resuspension buffer (NSB, 10mL DPBS + 10µl RNasin Plus), then centrifuged at 500g for 5 min at 4°C. This step was repeated and then the suspension was passed through a 5 µm strainer. The suspension was centrifuged at 500g for 5 min at 4°C, and the pellet was resuspended in 10 ml NSB. The nuclei suspension was mixed with 1 µl Vybrant Ruby stain and incubated on ice for 15 min. The nuclei were sorted in 500 µl final resuspension buffer (FSB, 1ml DPBS with 1% BSA + 5 µl Protector RNase inhibitor) with a low flow rate and pressure to ensure high viability. A 561+683 emission gate for the ruby stain was used to positively select nuclei from debris. Low trigger pulse width was used as singlet discriminator. One hundred thousand nuclei were targeted for collection and centrifuged at 500g for 5 min at 4°C. The top supernatant was carefully removed, and 50 µl volume was left to resuspend the

nuclei pellet. Ten microliters of nuclei were mixed with 10 µl Trypan Blue and then loaded on the Fuchs-Rosenthal disposable hemocytometer for counting. Nuclei were directly loaded to 10X chips for Gel Bead-In Emulsions (GEM) generation.

Single nuclear RNA sequencing (snRNAseq). The snRNA-Seq was performed using a Chromium Next GEM Single Cell 3′ Reagent Kit v3.1 (10x Genomics). Single nuclei were partitioned in droplets with single Gel Beads, which contained primers with cell-tagging indexes. Single nucleus suspensions were loaded targeting 10,000 nuclei per sample. The resulting cDNA was profiled on a Bioanalyzer NanoChip (Agilent), then used as a template for library preparation according to the reagent kit protocol. The final libraries were profiled on a Tapestation D1000 tape (Agilent) and quantified using real time PCR (Kapa Biosystems) on a StepOnePlus Real Time PCR workstation (Thermo/ABI). Libraries were sequenced on a NovaSeq 6000 (Illumina). FASTQ files were prepared using bcl2fastq (Illumina) and then aligned to a reference genome using Cellranger v. 7.1.0 (10x Genomics). Reads were mapped to both exonic and intronic regions to include the pre-mRNA transcriptome. Library preparation and sequencing were done by the OHSU Integrated Genomics Laboratory, RRID:SCR_022651.

***Single nuclear RNA sequencing transcriptional analysis*. Ambient RNA, doublet, and mitochondrial feature removal.** Ambient RNA contamination was estimated and removed using SoupX v. 1.6.2 (5). Cells with cleaned-up reads were then subjected to doublet removal using DoubletFinder v 2.0.3 (6). Mitochondrial features were manually removed by excluding all features starting with *mt- (7)*. These processing steps were done separately for each sample. Count matrices now only containing singlets were loaded to Seurat v. 4.3. for merging and further filtering (8). Nuclei with the features < 500, and counts > 20,000 were considered low quality nuclei and were filtered out. The filtered Seurat object was then inputted to SCTransform for normalization and variance stabilization (9). The normalized count matrix was integrated using Seurat’s built-in *FindIntegrationAnchors (8,10)*.

Batch effect correction, and cell clustering. Dimensional reduction analysis was performed using Principal Component Analysis (PCA). Clusters were generated using the built-in *FindNeighbors* and *FindClusters* Seurat commands employing the first 30 PCA dimensions with a 2.0 clustering resolution. The Uniform Manifold Approximation and Projection (UMAP) technique was used for 2D projection and visualization. Clusters were annotated based on selected canonical gene markers (11). The final Seurat object from 8 animals had a total of 80013 nuclei. The final annotated Seurat object used for all downstream analysis can be found at GSE253448 and online at https://nelsonlab.shinyapps.io/PTACE2KO_Kidney/.

SnRNA-seq differential gene analysis. Differential gene expression analysis was done with *FindMarkers* using the normalized and corrected values stored in the SCT slot employing the Wilcoxon Rank Sum test. The final differentially expressed gene list was filtered based on minimum detection rate (min.pct) at 10% and an adjusted p-value of 0.05. The list of DEGs for each population comparing PT ACE2 KO Saline compared to Control Saline and PT ACE2 KO AngII compared to Control AngII are included in **Supplemental Files 1 and 2,** respectively.

Pathway Analysis. Differentially expressed genes for proximal tubule cells (PTS1, PTS2, and PTS3) were subjected to overrepresentation analysis using the *enrichGo* function of *ClusterProfiler (12)*. Gene symbols were converted to ENTREZIDs using the Biological ID translator feature. Upregulated pathways were defined as pathways corresponding to the genes with actual log fold difference of > 1.25 and downregulated pathways with values < -1.25. The list of pathways for PT ACE2 KO Saline compared to Control Saline and PT ACE2 KO AngII compared to Control AngII are in **Supplemental File 3.** Pathways uniquely enriched in either comparison were examined, and those relating to transporter activity or angiotensin signaling were selected for visualization. Pathways related to brush border and cytoskeletal organization were enriched in both PT ACE2 KO Saline and AngII treatment groups relative to their Control counterparts. A list of genes queried for each Gene Ontology (GO) term analyzed in Figure 8 is

included in **Supplemental Table 2.** Genes queried for each Gene Ontology (GO) term analyzed in Supplemental Figure 4 are included in **Supplemental Table 3.**

Data availability: All snRNAseq data (FASTQ, Cell Ranger Outputs, and Seurat Object) have been uploaded to GEO (GSE253448, reviewer token: gzepkmgabhmhvqp) and bioinformatic code used for analysis and creation of figures has been published on github (https://github.com/JonathanWNelson/PTACE2KO). Additionally, the dataset is hosted online as a ShinyCell app (13) (https://nelsonlab.shinyapps.io/PTACE2KO_Kidney/) to allow exploration of the dataset through an online browser.

References

1. Su XT, Reyes JV, Lackey AE, et al. Enriched Single-Nucleus RNA-Sequencing Reveals Unique Attributes of Distal Convoluted Tubule Cells. *J Am Soc Nephrol*. Published online January 19, 2024. doi:10.1681/ASN.0000000000000297

2. Drokhlyansky E, Smillie CS, Van Wittenberghe N, et al. The Human and Mouse Enteric Nervous System at Single-Cell Resolution. *Cell*. 2020;182(6):1606-1622.e23. doi:10.1016/j.cell.2020.08.003

3. Wilson PC, Wu H, Kirita Y, et al. The single-cell transcriptomic landscape of early human diabetic nephropathy. *Proc Natl Acad Sci U S A*. 2019;116(39):19619-19625. doi:10.1073/pnas.1908706116

4. Kirita Y, Wu H, Uchimura K, Wilson PC, Humphreys BD. Cell profiling of mouse acute kidney injury reveals conserved cellular responses to injury. *Proc Natl Acad Sci U S A*. 2020;117(27):15874-15883. doi:10.1073/pnas.2005477117

5. Young MD, Behjati S. SoupX removes ambient RNA contamination from droplet-based single-cell RNA sequencing data. *Gigascience*. 2020;9(12):giaa151. doi:10.1093/gigascience/giaa151

6. McGinnis CS, Murrow LM, Gartner ZJ. DoubletFinder: Doublet Detection in Single-Cell RNA Sequencing Data Using Artificial Nearest Neighbors. *Cell Syst*. 2019;8(4):329-337.e4. doi:10.1016/j.cels.2019.03.003

7. Schirmer L, Velmeshev D, Holmqvist S, et al. Neuronal vulnerability and multilineage diversity in multiple sclerosis. *Nature*. 2019;573(7772):75-82. doi:10.1038/s41586-019-1404-z

8. Hao Y, Hao S, Andersen-Nissen E, et al. Integrated analysis of multimodal single-cell data. *Cell*. 2021;184(13):3573-3587.e29. doi:10.1016/j.cell.2021.04.048

9. Hafemeister C, Satija R. Normalization and variance stabilization of single-cell RNA-seq data using regularized negative binomial regression. *Genome Biol*. 2019;20(1):296. doi:10.1186/s13059-019-1874-1

10. Satija R, Farrell JA, Gennert D, Schier AF, Regev A. Spatial reconstruction of single-cell gene expression data. *Nat Biotechnol*. 2015;33(5):495-502. doi:10.1038/nbt.3192

11. Balzer MS, Rohacs T, Susztak K. How Many Cell Types Are in the Kidney and What Do They Do? *Annu Rev Physiol*. 2022;84:507-531. doi:10.1146/annurev-physiol-052521-121841

12. Yu G, Wang LG, Han Y, He QY. clusterProfiler: an R package for comparing biological themes among gene clusters. *OMICS*. 2012;16(5):284-287. doi:10.1089/omi.2011.0118

13. Ouyang JF, Kamaraj US, Cao EY, Rackham OJL. ShinyCell: simple and sharable visualization of single-cell gene expression data. *Bioinformatics*. 2021;37(19):3374-3376. doi:10.1093/bioinformatics/btab209

Supplemental Table 1. Sodium transporter immunoblot loading and antibody information

Antibody Target	~kDa	Protein/ lane cortex (μg)	Protein/ lane medulla (μg)	Primary antibody supplier	Ab host	Dilution	Time	Secondary antibody supplier	Host and target	Dilution	Time
ACE1	195	5, 2.5	NA	Santa Cruz (sc-12187)	Gt	1:1000	O/N	Invitrogen	DAG 680	1:5000	1 hr
ACE2	100	20, 10	NA	Abcam (ab108252)	Rb	1:2000	O/N	Invitrogen	GAR 680	1:5000	1 hr
Agt	50	8	NA	Abcam (ab213705)	Rb	1:2000	O/N	Invitrogen	GAR 680	1:5000	1 hr
AQP1	35<br>23	2, 1	2, 1	AlphaDiag. Int'l (AQP11-A)	Rb	1:5000	2 hr	LI-COR	GAR 800	1:5000	1 hr
AQP2	37<br>23	2, 1	2, 1	Santa Cruz (sc-515770)	Mu	1:1000	2 hr	Invitrogen	GAM 680	1:5000	1 hr
AQP2pS256	37<br>23	20, 10	10, 5	ThermoFisher (PA5-38407)	Rb	1:1000	2 hr	Invitrogen	GAR 680	1:5000	1 hr
Claudin-2	23	5, 2.5	NA	Thermo (32-5600)	Mu	1:2000	O/N	LI-COR	GAM 800	1:5000	1 hr
Claudin-8	23	20, 10	NA	Thermo (40-0700Z)	Rb	1:2000	O/N	Invitrogen	GAR 680	1:5000	1 hr
ENaC-γ<br>Full-length<br>Cleaved	 ~80<br>~60	40, 20	NA	Loffing (Zurich)	Rb	1:15000	O/N	Invitrogen	GAR 680	1:5000	1 hr
Na,K-ATPase α1	100	1, 0.5	2, 1	Kashgarian (Yale)	Mu	1:200	2 hr	LI-COR<br>Invitrogen	GAM 800<br>GAM 680	1:5000	1 hr
Na,K-ATPase β1	50	10, 5	10, 5	Martín-Vasallo (ULL)	Rb	1:2000	O/N	Invitrogen	GAR 680	1:5000	1 hr
NCC	160	40, 20	NA	McDonough	Rb	1:5000	O/N	Invitrogen	GAR 680	1:5000	1 hr
NCCpT53	160	40, 20	NA	Loffing (Zurich)	Rb	1:5000	2 hr	Invitrogen	GAR 680	1:5000	1 hr
NHE3	~83	40, 20	10	McDonough	Rb	1:2000	O/N	Invitrogen	GAR 680	1:5000	1 hr
NKCC2	160	20, 10	10, 5	DSHB (Iowa)	Mu	1:6000	O/N	LI-COR	GAM 800	1:5000	1 hr
NKCC2-pT96T101	160	20, 10	10, 5	Forbush (Yale)	Rb	1:2000	2 hr	Invitrogen	GAR 680	1:5000	1 hr
SGLT2	70	20, 10	NA	Koepsell (Würzburg)	Rb	1:5000	2 hr	Invitrogen	GAR 680	1:5000	1 hr

Ab, antibody; Agt, angiotensinogen; AQP1/2, aquaporin 1 and 2; AQP2pS256, AQP2 phosphorylated serine 256; ENaC, epithelial sodium channel; kDa, kilodalton; NCC, sodium-chloride co-transporter; NCCpT53, NCC phosphorylated threonine 53; NHE3, sodium hydrogen exchanger 3; NKCC2, sodium potassium chloride cotransporter; NKCC2pT96T101, NKCC2 phosphorylated threonine 96 and 101; SGLT2, sodium glucose co-transporter; Gt, goat; Rb, rabbit; mu, mouse; O/N, overnight; DAG, donkey anti-goat; GAR, goat anti-rabbit; GAM, goat anti-mouse.

Supplemental Table 2. Circulating renin angiotensin system (RAS) metabolites at day 5 of AngII treatment

	Ang(1-10) (ng/mL)	Ang(1-9) (ng/mL)	Ang(1-8) (ng/mL)	Ang(1-7) (ng/mL)	Ang(2-8) (ng/mL)	Ang(3-8) (ng/mL)	Ang(1-9)/ (Ang(1-10) (ng/mL)
**Control**	0.48 ± 0.13	8.73 ± 9.45	5.82 ± 2.53	0.72 ± 0.19	5.40 ± 0.13	0.77 ± 0.10	22.57 ± 24.86
**PT ACE2 KO**	0.44 ± 0.03	1.44 ± 0.60	6.63 ± 4.87	0.62 ± 0.07	5.37 ± 0.13	0.90 ± 0.13	3.27 ± 1.20
p value	0.28	0.60	0.45	0.18	0.35	0.05	0.08

Assessment of RAS metabolites using mass spectrometry (Control n=4, PT ACE2 KO n=4). RAS metabolites were analyzed by t-test and expressed as mean ± SEM.

Supplemental Table 3. Functional assessments of electrolyte excretion in proximal tubule-specific ACE2 knockout mice

	Control	PT ACE2 KO	*p* value
**Number of animals**	6	9	
**UNaV, mmol/24 h**	0.17 ± 0.05	0.22 ± 0.04	0.14
**UKV, mmol/24 h**	0.29 ± 0.06	0.35 ± 0.07	0.11
**Na^+ balance, mmol**	0.0026 ± 0.04	-0.029 ± 0.03	0.14

Assessments of electrolyte excretion in response to acute sodium challenge after 5 days of continuous AngII infusion (1000 ng/kg/min). UnaV, urinary sodium excretion, UKV, urinary potassium excretion. Values calculated as the mean ± standard deviation and were analyzed as a *t*-test between Control and PT ACE2 KO groups.

Supplemental Table 4. Genes assessed in gene ontology pathway analysis

Comparison	GO ID	GO term	q value	Genes assayed
**PT ACE2 KO Saline vs. Control Saline**	GO:0015078	proton transmembrane transporter activity	2.2021E-06	Slc25a5/ Uqcrfs1/ Atp5d/ Atp5b/ Atp6v0a4/ Cox4i1/ Cox5a/ Cyc1/ Slc9a3
	GO:0032836	glomerular basement membrane development	0.03451861	Col4a4/ Col4a3
	GO:1902495	transmembrane transporter complex	0.02681345	Kcnma1/ Ndufa4/ Uqcrfs1/ Ndufb8/ Fxyd2/ Uqcrq/ Cyc1
	GO:1902600	proton transmembrane transport	8.1789E-05	Ndufa4/ Atp5d/ Taco1/ Atp5b/ Atp6v0a4/ Fxyd2/ Slc9a3
	GO:1990351	transporter complex	0.03180501	Kcnma1/ Ndufa4/ Uqcrfs1/ Ndufb8/ Fxyd2/ Uqcrq/ Cyc1
	GO:0015824	proline transport	0.0265505	Slc1a4/ Slc6a15/ Ace2
	GO:1904383	response to sodium phosphate	0.0001977	Enpp1/ Abcc6/ Fgfr3/ Fgfr1/ Runx2
	GO:0034763	negative regulation of transmembrane transport	0.0291969	Irs2/ Kcnrg/ Trdn/ Enpp1/ Bcl2/ Hecw2/ Ppp3ca/ Inpp5k/ Oaz2
	GO:0071248	cellular response to metal ion	0.0117904	Bnip3/ Aoc1/ Slc40a1/ Itpkb/ Gabrb3/ Aqp1/ Lgmn/ Asph/ Slc1a1/ Tfrc/ Slc12a2
	GO:0030512	negative regulation of transforming growth factor beta receptor signaling pathway	0.0258875	Pbld2/ Il17rd/ Ldlrad4/ Hdac1/ Xbp1/ Htra1/ Smurf1
**PT ACE2 KO AngII vs. Control AngII**	GO:0015081	sodium ion transmembrane transporter activity	0.00210404	Mfsd2a/ Slc6a18/ Slc28a1/ Slc6a15/ Slc18a1/ Slc20a1/ Slc5a10/ Slc5a1/Slc5a8/ Slc23a1/ Slc9a8/ Slc5a4a
	GO:0015296	monoatomic anion: monoatomic cation symporter activity	0.02322034	Slc39a14/ Slc6a18/ Slc12a6/ Slc18a1
	GO:0015347	sodium-independent organic anion transmembrane transporter activity	0.00212968	Slco1a1/ Slco3a1/ Slc22a30/ Slco1a6/ Slc22a28
	GO:0015370	solute: sodium symporter activity	2.156E-05	Mfsd2a/ Slc6a18/ Slc28a1/ Slc6a15/ Slc18a1/ Slc20a1/ Slc5a10/ Slc5a1/Slc5a8/ Slc23a1/ Slc5a4a
	GO:0032414	positive regulation of ion transmembrane transporter activity	0.03867606	Cacnb4/ Glrx/ Ppp3cc/ Vmp1/ Adrb2/ Nedd4l/ Stim2/ Stim1/ Ndufa4
	GO:0010958	regulation of amino acid import across plasma membrane	0.02616598	Cltrn/ Slc43a2/ Ace2
	GO:0015175	neutral L-amino acid transmembrane transporter activity	0.01041106	Slc43a2/ Slc7a8/ Slc6a19/ Sfxn2/ Slc7a9
	GO:0022898	regulation of transmembrane transporter activity	0.03294751	Abcb1b/ Cltrn/ Trdn/ Pde4b/ Chrna4/ Akap7/ Camk2d/ Cnksr3/ Ace2/ Ppargc1a/ Ptpn3/ Plcb1
	GO:0070293	renal absorption	0.00253007	Wnk4/ Aqp1/ Klhl3/ Slc5a2/ Amn
	GO:0035725	sodium ion transmembrane transport	0.00253007	Wnk4/ Slc6a19/ Camk2d/ Cnksr3/ Slc13a3/ Slc5a2/ Tpcn1/ Slc34a1/ Ptpn3/ Slc6a8/ Plcb1

Comparison of enriched pathways unique to either Saline- or AngII-treated PT ACE2 KO kidneys relative to their Control counterparts. q value represents the minimum false discovery rate at which an observed score is deemed significant; analogous to the p value incorporating multiple testing corrections. GO ID, gene ontology identification.

Supplemental Table 5. Genes assessed in gene ontology pathway analysis of the proximal tubule brush border

Comparison	GO ID	GO term	q value	Genes assayed
**PT ACE2 KO vs. Control**	GO:0005903	brush border	Saline: 0.01025519; AngII: 3.8479E-16	Slc22a12/ Myh10/ Eps8/ Car4/ Aqp1/ Myh14/ Slc11a2/ Lrp2/ Ace2/ Myo1e/ Gna12/ Prom1/ Cltrn/ Enpep/ Lima1/ Abcc2/ Pth1r/ Slc6a19/ Folr1/ Shank2/ Slc5a2/ Amn/ Add3/ Slc34a1/ Slc7a9/ Abcg2/ Slc2a2/ Myo1d/ Myo18a/ Slc22a5
	GO:0016324	Apical Plasma Membrane	Saline: 8.3175E-06; AngII: 3.6711E-20	Slc10a2/ Abcb1b/ Slc22a12/ Nrg1/ Cyp4a12a/ Slc44a4/ Slc39a8/ Pld1/ Abcc4/ Abcc6/ Mpdz/ Car4/ Aqp1/ Nox4/ Cyp4a10/ Shroom3/ Slc11a2/ Itpk1/ Stx3/ Lrp2/ Ace2/ Slc16a2/ Slc2a5/ Slc1a1/ Slc12a2/ Msn/ Dpp4/ Cyp4a14/ Prom1/ Slc22a8/ Enpep/ Abcc2/ Pth1r/ Slc7a8/ Slc13a1/ Atp2b1/ Cyba/ Akap7/ Slc6a19/ Folr1/ Car12/ Shank2/ Slc5a2/ Amn/ Slc17a1/ Pard3b/ Erbb3/ Slc34a1/ Slc7a9/ Shroom4/ Abcg2/ Prkaa2/ Slc2a2/ Ctsb/ Akr1a1/ Ctnnb1/ Slc6a8/ Slc22a5
	GO:0031253	Cell projection membrane	Saline: 0.0325931; AngII: 1.5435E-14	Slc22a12/ Eps8/ Sh3yl1/ Car4/ Efcab7/ Aqp1/ Pde6a/ Iqce/ Slc11a2/ Lrp2/ Plekha1/ Ace2/ Gna12/ Bbs7/ Inpp5k/ Msn/ Prom1/ Cltrn/ Enpep/ Lima1/ Abcc2/ Pth1r/ Slc7a8/ Gabarapl1/ Atp2b1/ Slc6a19/ Folr1/ Spata13/ Ptprj/ Shank2/ Appl2/ Slc5a2/ Amn/ Epb41l3/ Cask/ Slc34a1/ Slc7a9/ Abcg2/ Insr/ Slc22a2/ Myo1d/ Ctnnb1/ Slc22a5
	GO:0032956	regulation of actin cytoskeleton organization	Saline: 0.02319886; AngII: 0.00138714	Cit/ Fhod3/ Eps8/ Fmn1/ Nrp1/ Fchsd2/ Evl/ Nox4/ Smad3/ Dixdc1/ Serpinf2/ Arhgap18/ Alms1/ Cgnl1/ Inpp5k/ Limch1/ Id1/ Lima1/ Cotl1/ Add3/ Cyfip2/ Baiap2/ Phldb2/ F11r/ Cd2ap/ Arhgap35/ Tsc1
	GO:0098862	cluster of actin-based cell projections	Saline: 0.00044559; AngII: 1.2313E-13	Slc22a12/ Myh10/ Eps8/ Dcdc2a/ Clic5/ Fchsd2/ Car4/ Aqp1/ Myh14/ Slc11a2/ Lrp2/ Ace2/ Myo1e/ Gna12/ Dock4/ Zswim6/ Prom1/ Cltrn/ Enpep/ Lima1/ Abcc2/ Pth1r/ Slc6a19/ Folr1/ Shank2/ Slc5a2/ Amn/ Add3/ Slc34a1/ Slc7a9/ Abcg2/ Slc2a2/ Myo1d/ Myo18a/ Slc22a5
	GO:0110053	regulation of actin filament organization	Saline: 0.00843107; AngII: 0.00083548	Cit/ Fhod3/ Eps8/ Fmn1/ Nrp1/ Fchsd2/ Evl/ Nox4/ Smad3/ Serpinf2/ Arhgap18/ Alms1/ Cgnl1/ Inpp5k/ Limch1/ Id1/ Lima1/ Cotl1/ Add3/ Cyfip2/ Baiap2/ Phldb2/ F11r/ Arhgap35/ Tsc1

Pathways related to brush border and cytoskeletal organization were enriched in both PT ACE2 KO Saline and AngII kidneys relative to their Control counterparts. q value represents the minimum false discovery rate at which an observed score is deemed significant; analogous to the p value incorporating multiple testing corrections. GO ID, gene ontology identification.

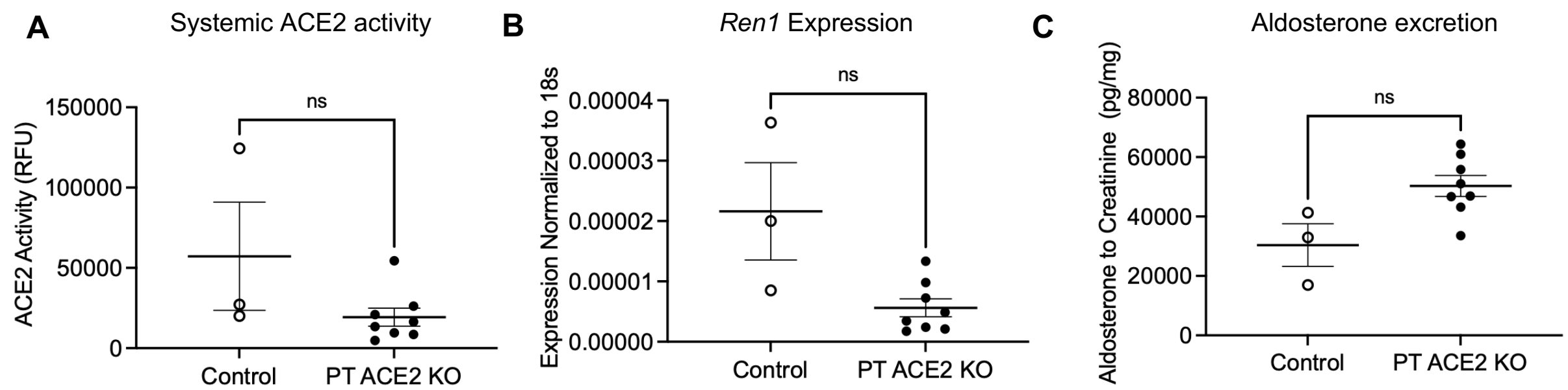

Supplemental Figure 1. Loss of ACE2 from the proximal tubule is restricted to the intrarenal RAS. A. Systemic ACE2 activity, **B.** Kidney *Ren1* mRNA expression, and **C.** urine aldosterone excretion from PT ACE2 KO and Control mice after 5 days of AngII infusion (1000 ng/kg/min; Control n=3, PT ACE2 KO n=8). Systemic ACE2 activity, Ren1 expression, and aldosterone excretion were analyzed as a t-test with Welch correction. Results are expressed as mean ± SEM.

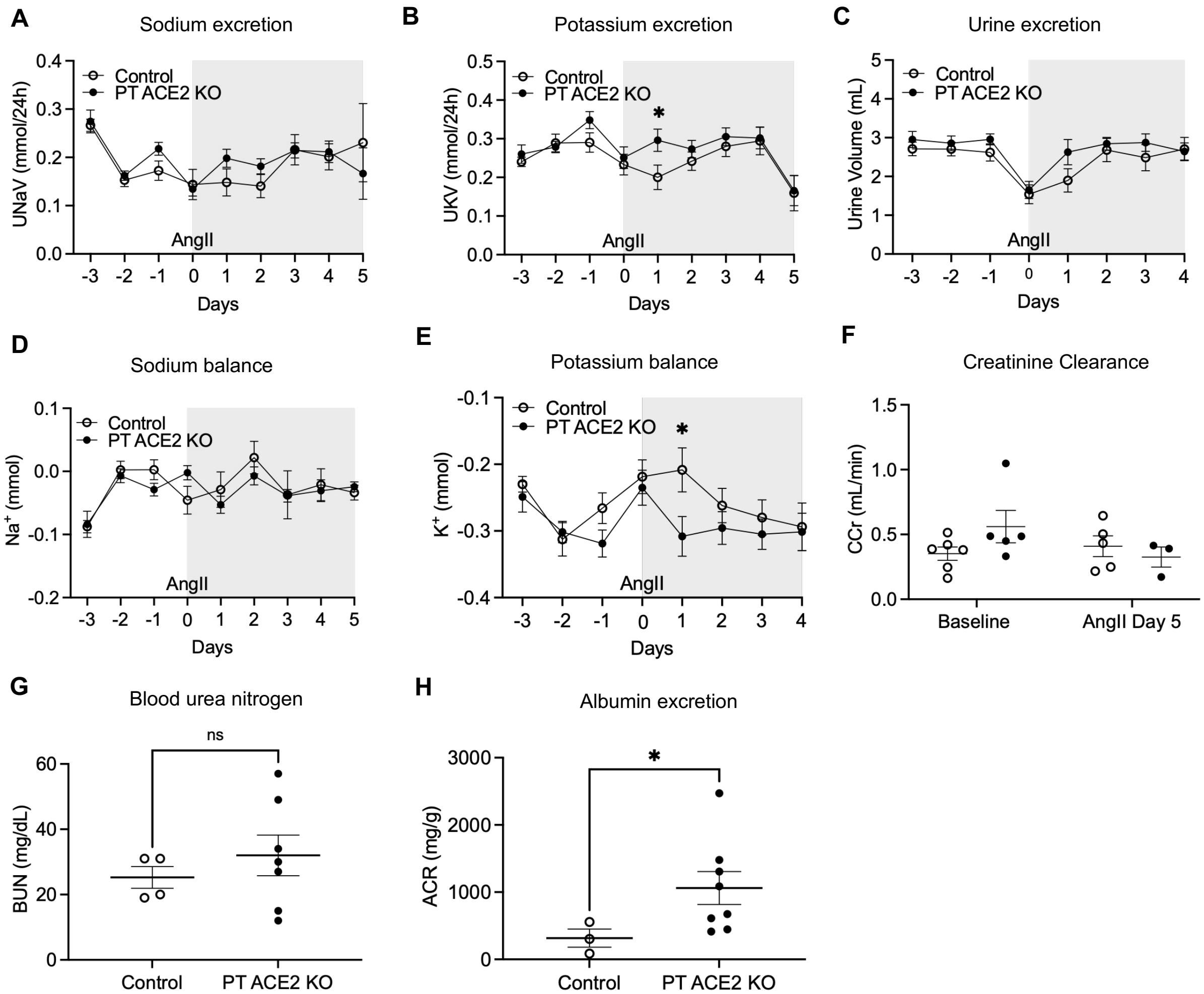

Supplemental Figure 2. Intra-renal responses support sodium and potassium homeostasis in PT ACE2 KO mice. A. Balance study sodium **B.** potassium, and **C.** urine excretion of Control and PT ACE2 KO mice at baseline and during chronic AngII infusion (1000 ng/kg/min). Mice were housed in individual metabolic cages with free access to gel diet (Control n=6, PT ACE2 KO n=12). **D.** Sodium and **E.** potassium balance. **F.** Creatinine clearance at baseline and with AngII infusion (Control n=5-6, PT ACE2 KO n=3-5). **G.** Blood urea nitrogen (BUN) measured on day 5 of AngII treatment (Control n=4, PT ACE2 KO n=7). **H.** Albumin excretion measured as albumin-to-creatinine ratio (ACR; Control n=3, PT ACE2 KO n=8). Balance study and creatinine clearance data were analyzed as two-way ANOVA with Tukey's multiple comparison post hoc analysis. BUN and ACR were analyzed as a t-test with Welch's correction. Results are expressed as mean ± SEM; *p<0.05

A

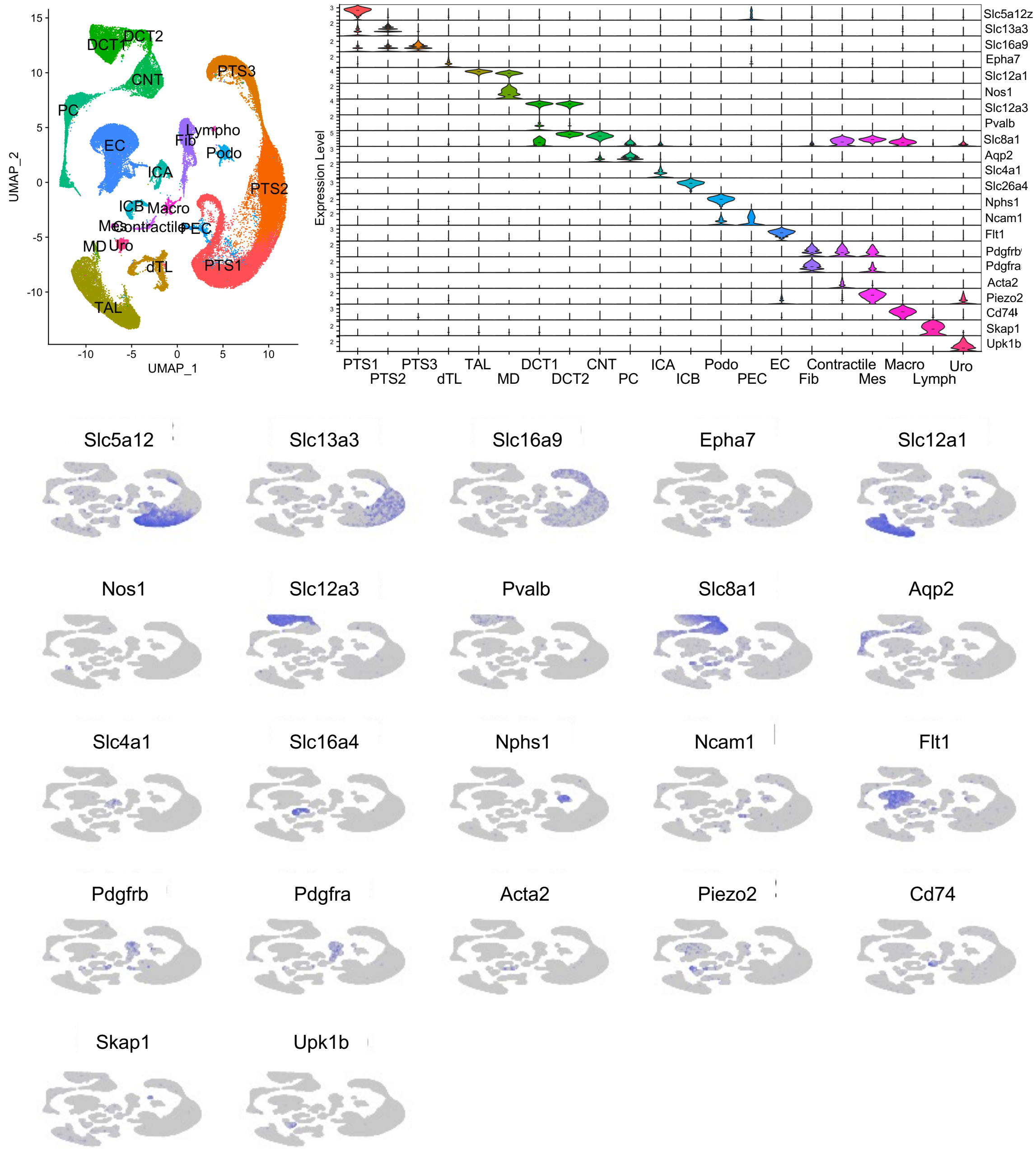

Supplemental Figure 3. Canonical marker expression for all cell types. A. UMAP projection for all nuclei after initial pre-processing and filtering and violin plots for canonical markers of different cell types. **B.** UMAP projection for canonical markers.

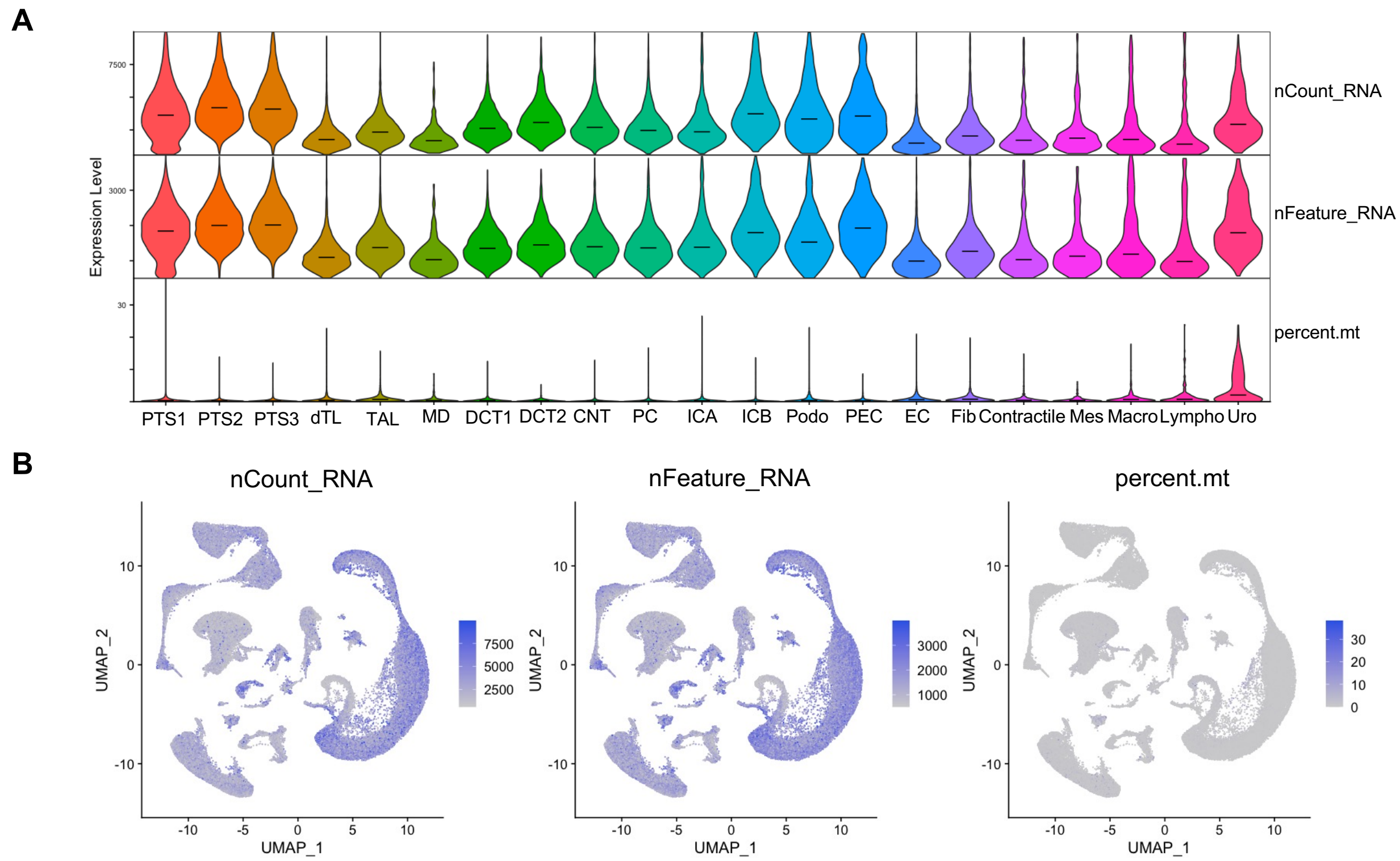

Supplemental Figure 4. Quality control of all nuclei. A. Violin plots for nCount_RNA (number of RNA counts), nFeature_RNA (number of features), and percent.mt (mitochondria RNA percentage). **B.** UMAP projection for nCount_RNA, nFeature_RNA, and percent.mt

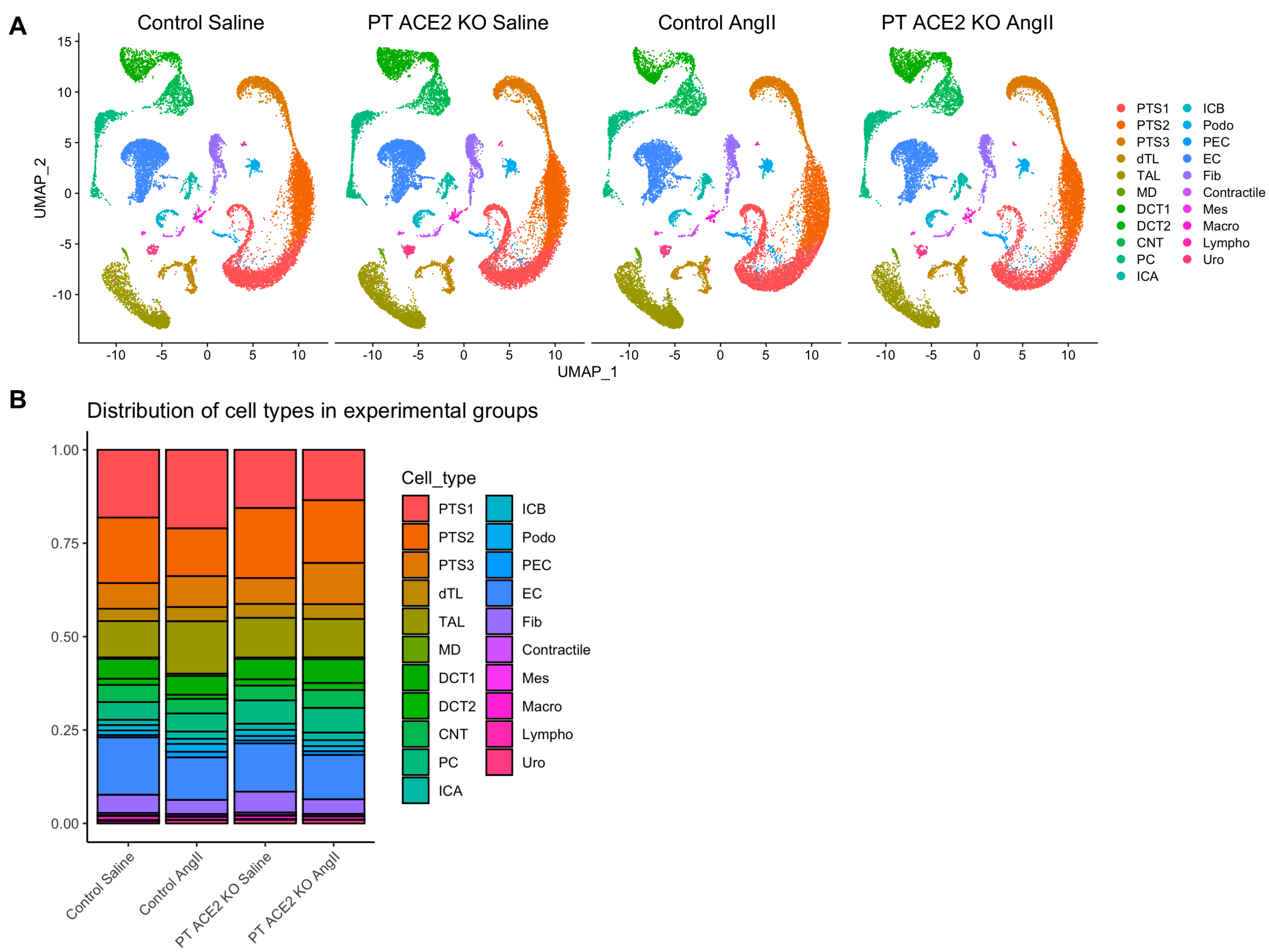

Supplemental Figure 5. Representation of cell types across experimental groups. A. UMAP projection all nuclei in Control Saline, PT ACE2 KO Saline, Control AngII, PT ACE2 KO AngII groups. **B.** Distribution of cell types across all experimental groups.

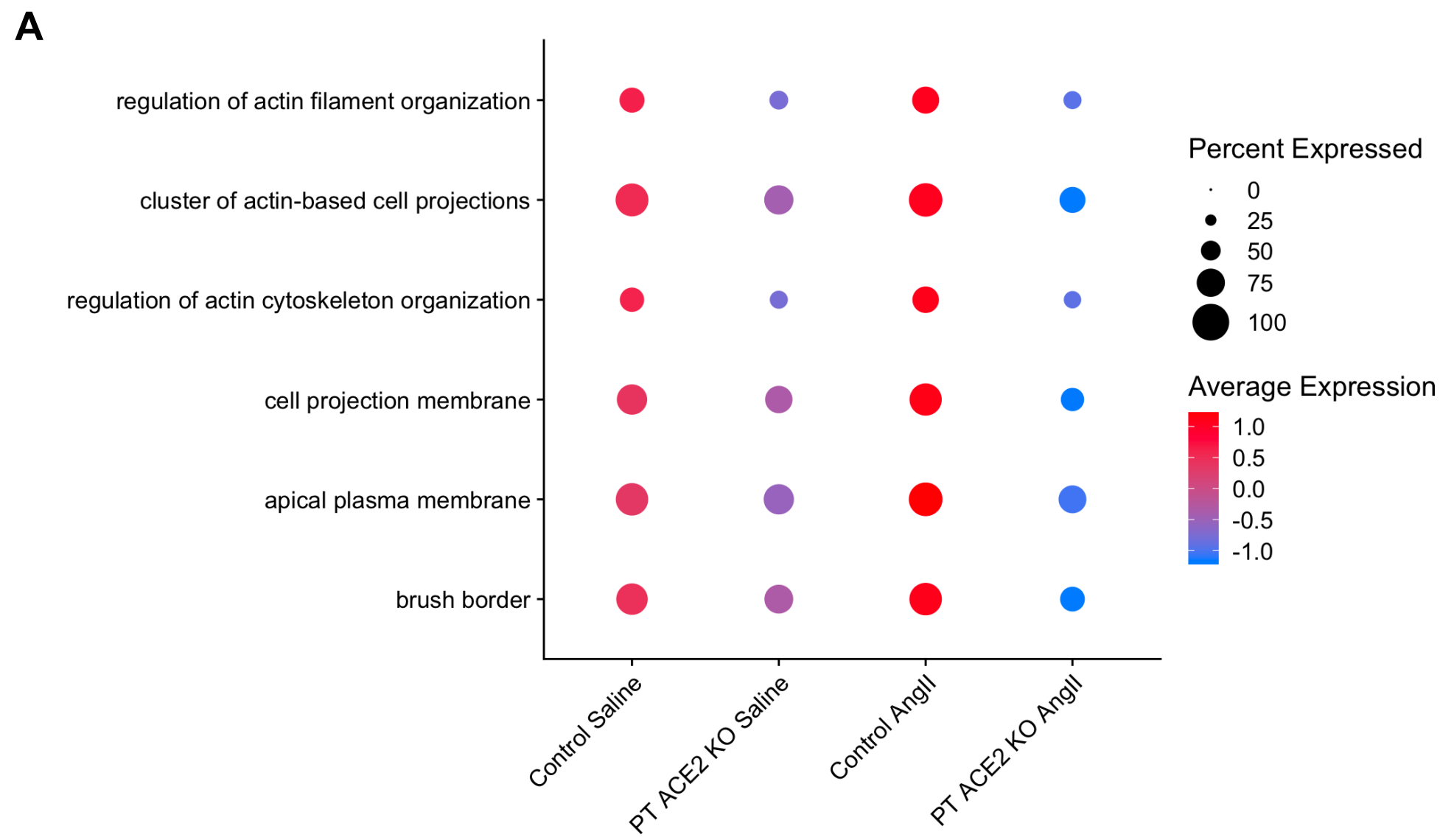

Supplemental Figure 6. Loss of ACE2 from the proximal tubule alters the transcriptional profile of the apical brush border. A. Enrichment of pathways found significant in PT ACE2 KO Saline vs. Control Saline and PT ACE2 KO AngII vs. Control AngII.

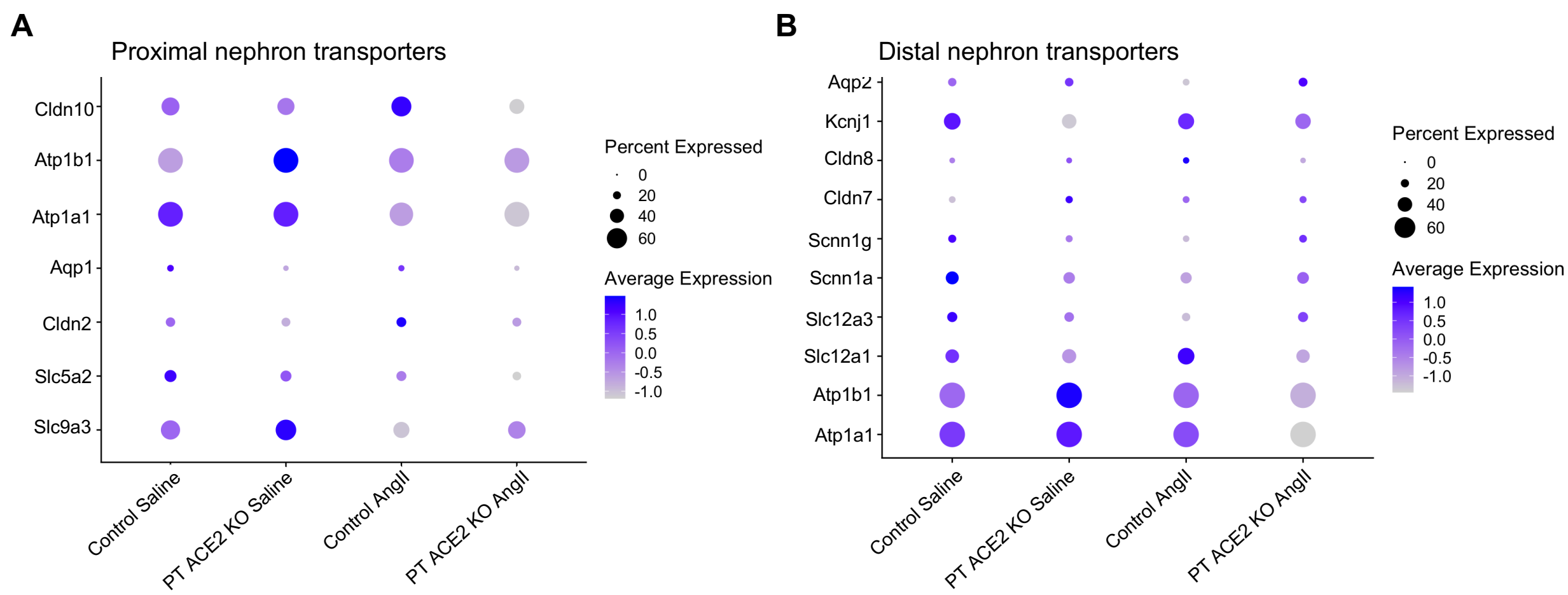

Supplemental Figure 7. Transcriptional profile of key sodium transporters and intrarenal RAS components. A. Expression of transporter genes along the proximal nephron clusters (proximal tubule segments 1-3 and the thick ascending limb). **B.** Expression of transporter genes along the distal nephron clusters (thick ascending limb, macula densa, distal convoluted tubule 1 and 2, connecting tubule, A and B type intercalated cells, and urothelium).
